# Allosteric communication mechanism in the glucagon receptor

**DOI:** 10.1016/j.jbc.2025.108530

**Published:** 2025-04-23

**Authors:** Wijnand J.C. van der Velden, Elizaveta Mukhaleva, Nagarajan Vaidehi

**Affiliations:** Department of Computational and Quantitative Medicine, Beckman Research Institute of the City of Hope, Duarte, California, USA

**Keywords:** glucagon, glucagon receptor, G protein–coupled receptor, molecular dynamics, allosteric communication, SNP, class B1, G protein, diabetes, metabolic disorders

## Abstract

Recent drug development suggests agonists may be more promising against glucagon receptor dysregulation in metabolic disorders. Allosteric modulation may pave an alternative way to initiate responses that are required to target these metabolic disorders. Here, we investigated the allosteric communication mechanisms within the glucagon receptor using molecular dynamics simulations on five receptor states. Results highlighted that the extracellular domain is dynamic in the absence of an orthosteric agonist. In the presence of a partial agonist, we observed increased flexibility in the N terminus of the receptor compared with the full agonist–bound receptor. Class B1 G protein–coupled receptor (GPCR) microswitches showed repacking going from the inactive state to the active state, allowing for G protein coupling. In the full agonist– and G protein–bound state, Gα_s_ showed both translational and rotational movement in the N terminus, core, and α5-helix, thereby forming key interactions between the core of the G protein and the receptor. Finally, the allosteric communication from the extracellular region to the G protein coupling region of the receptor was the strongest in the intracellular negative allosteric modulator–bound state, the full agonist and G protein–bound state, and the full agonist–bound G protein–free state. The residue positions predicted to play a significant role in the allosteric communication mechanism showed overlap with disease-associated mutations. Overall, our study provides insights into the allosteric communication mechanism in a class B1 GPCR, which sets the foundation for future design of allosteric modulators targeting the glucagon receptor.

Glucagon is a 29-amino acid peptide hormone with its secretory origin found in the ɑ-cells of the pancreatic islets (1, 2). Its primary function involves glucose production from hepatocytes in the liver through glycogenolysis and gluconeogenesis (3). Besides glucose production, it also has implications in hepatic amino acid and lipid metabolism where it shows a negative feedback loop toward glucagon secretion (4, 5, 6, 7). Hence, the liver–α-cell axis, which is the interplay between the pancreas and liver, seems to be critical in glucose production and amino acid and lipid metabolism (8, 9).

The glucagon receptor is a class B1 G protein–coupled receptor (GPCR) that is not only mainly expressed on hepatocytes but also shown to be present on β-cells and δ-delta cells of the pancreatic islets, kidneys, and heart (10, 11, 12). Once bound to its endogenous ligand, glucagon, the receptor couples to one of several heterotrimeric G proteins, including Gα_s_, Gα_i_, and Gα_q_, with Gα_s_ having the strongest affinity and efficiency (13). However, the coupling of all three Gα protein subtypes to the glucagon receptor has been shown to stimulate hepatic glucose production (14, 15, 16, 17).

Dysregulation of the glucagon receptor is associated with hyperglucagonemia, hyperaminoacidemia, and ɑ-cell hyperplasia (18). Correspondingly, these pathophysiological phenomena have been observed in patients who are suffering from diabetes, hepatic steatosis, cirrhosis, or chronic kidney disease (8, 19, 20, 21, 22, 23). Moreover, genetic variants of the glucagon receptor are linked to altered receptor function as well as selectivity toward G protein coupling (24). Currently, several missense variants or SNPs have been associated with metabolic disorders and tested *in vitro*, including G40^ECD^S, D63^ECD^N, P86^ECD^S, V368^6.59^M, and the double variant R225^3.30^H–V368^6.59^M (GPCRdb class B1 nomenclature in superscript) (25, 26, 27, 28, 29, 30, 31, 32, 33, 34). This makes the glucagon receptor an attractive target for therapeutic intervention.

Recent research has shown that glucagon receptor agonism (in combination with glucagon-like peptide 1 receptor [GLP-1R] agonism) rather than antagonism is more promising as a therapeutic intervention of combating diabetes and other metabolic disorders (35, 36, 37, 38). Hence, glucagon receptor agonism facilitates antidiabetic as well as antiobesogenic effects despite hyperglycemic properties; (a) stimulates β-cell-mediated insulin through glucose-dependent and -independent mechanisms, (b) enhances energy expenditure while suppressing food intake, and (c) reduces hepatic lipid accumulation, increases hepatic ureagenesis, improves systemic lipid metabolism, and circulating amino acid profiles (39).

Most drugs targeting class B1 GPCRs bind to the orthosteric binding site. However, given the therapeutic advantages of allosteric modulators (40), several positive allosteric modulators and negative allosteric modulators (NAMs) have been identified for class B1 GPCRs, encompassing a range that includes antibodies, receptor activity–modifying proteins, and small molecules (11, 41, 42, 43, 44, 45). For the glucagon receptor, two small-molecule NAMs (MK-0893 and NNC0640) were cocrystalized with the receptor (46, 47), bringing insight into binding sites of NAMs. Also, recently a conserved positive intracellular agonist was identified for class B1 GPCRs that acts on seven of the 15 class B1 GPCRs, including the glucagon receptor (48, 49). As this information shed light on conserved activation of class B1 receptor activation, selectivity concerns and off-target effects may arise when this compound would be used therapeutically. Hence, there is a requisite for selective glucagon receptor and dual GLP-1R/glucagon receptor small-molecule positive allosteric modulators as currently none are available.

Allosteric modulators have several therapeutic advantages over orthosteric ligands because of their potential to be target specific including specificity to disease-associated mutants. Computational methods that consider the receptor dynamics for identifying cryptic binding pockets combined with protein allostery play a critical role in identifying allosteric modulators (50). To design allosteric modulators for the glucagon receptor, this study is focused on uncovering the dynamics and the allosteric communication mechanism in the glucagon receptor in various active and inactive conformation states. Such mechanisms are not obvious from the analysis of static 3D structures.

## Results

**Figure 1:**
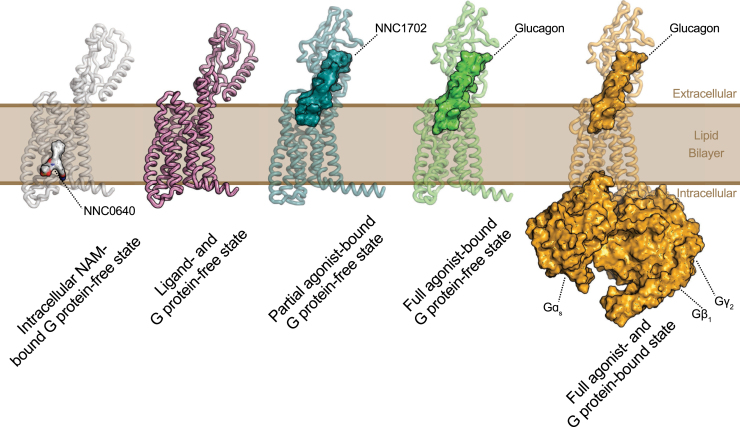
**Computationally simulated systems employed in the present study.** From *left to right*: intracellular NAM–bound (NNC0640) G protein–free inactive state (Protein Data Bank [PDB] accession number: 5XEZ); ligand– and G protein–free inactive state (PDB accession number: 5XEZ); the partial agonist–bound (NNC1702) G protein–free intermediate state (PDB accession number: 5YQZ); full agonist–bound (glucagon) G protein–free intermediate state (PDB accession number: 6LMK); full agonist– (glucagon) and G protein–bound (Gɑ_s_–Gβ_1_–Gγ_2_) fully active state (PDB accession number: 6LMK). NAM, negative allosteric modulator.

We performed all-atom molecular dynamics (MD) simulations of the glucagon receptor in a POPC (1-palmitoyl-2-oleoyl-*sn*-glycero-3-phosphocholine) lipid bilayer in the intracellular NAM–bound (NNC0640) G protein–free inactive state (Protein Data Bank [PDB] accession number: 5XEZ) (47), ligand- and G protein–free inactive state (PDB accession number: 5XEZ) (47), partial agonist–bound (NNC1702) G protein–free intermediate state (PDB accession number: 5YEZ) (51), full agonist–bound (glucagon) G protein–free intermediate state (PDB accession number: 6LMK) (52), and full agonist–(glucagon) and G protein–bound (Gɑ_s_–Gβ_1_–Gγ_2_) active state (PDB accession number: 6LMK) (52) (Fig. 1). For each system, we performed five independent MD simulation runs each 1000 ns long and utilized the full concatenated trajectory of 5 μs for analysis (see the Experimental procedures section for more details). However, for the full agonist–bound (glucagon) G protein–free state, we ran five 5000 ns unbiased simulations to allow for transition from the active state to an intermediate state. Since run 2 did not transition toward the intermediate state (measured as the transmembrane [TM] domain 3–TM6 distance; Fig. S1), we omitted this trajectory for further analysis. The last 1.5 μs of each of the other runs were used for final analysis as the RMSD of glucagon backbone and glucagon receptor backbone were most stable (*i.e.*, 6 μs in total, Fig. S2).

### The extracellular domain (ECD) of the glucagon receptor is highly dynamic in the intracellular NAM–bound and ligand-free states

**Figure 2:**
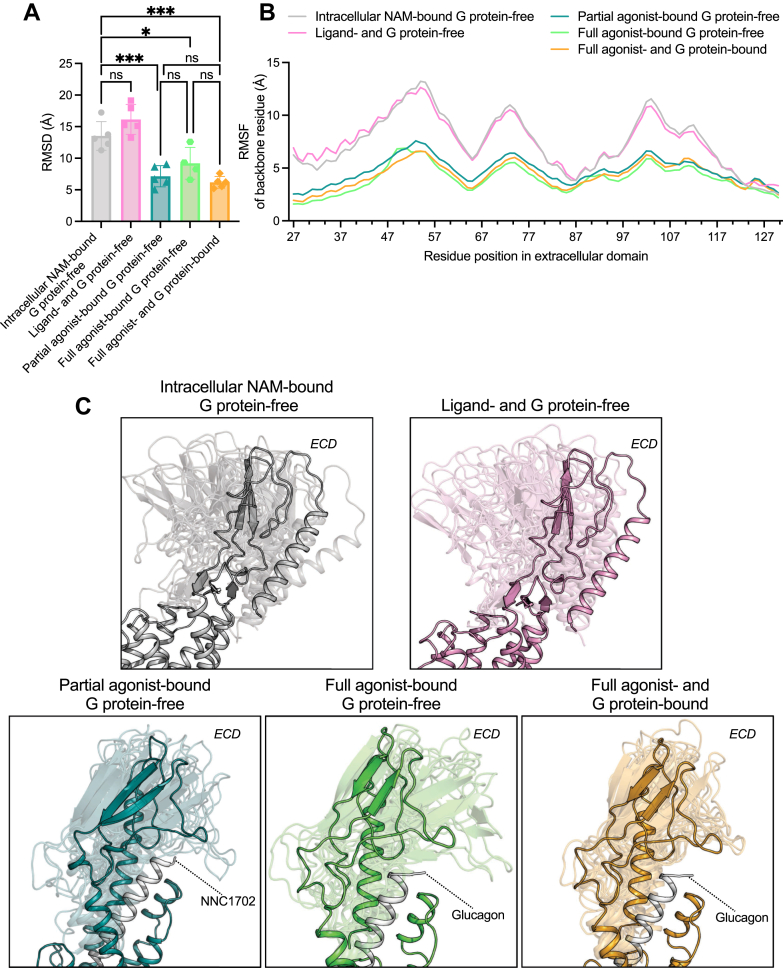
**Dynamics of the extracellular domain (ECD) in the glucagon receptor.***A*, RMSD of the ECD during MD simulations of the intracellular NAM–bound G protein–free, ligand– and G protein–free, partial agonist–bound G protein–free, full agonist–bound (glucagon) G protein–free, and full agonist– (glucagon) and G protein–bound (Gɑ_s_–Gβ_1_–Gγ_2_) states of the glucagon receptor with reference to the ECD of the starting structure of the glucagon receptor. *B*, root-mean-square fluctuation (RMSF) of individual ECD residues during MD. *C*, representative snapshots from the most populated conformational cluster extracted from MD simulations of the different glucagon receptor states overlayed with the experimental starting structure. The snapshots were taken from each of the five independent simulation replicates after 500 ns and 1000 ns of simulation time (10 in total per receptor state). For the full agonist–bound (glucagon) G protein–free state, snapshots were taken at 4250 ns and 5000 ns simulation time (eight in total). Data represent the mean ± SD of five (four for the full agonist–bound [glucagon] G protein–free state) independent simulation replicates in *A*, whereas they represent the mean in *B*, statistical significance was assessed using a one-way ordinary ANOVA with Dunnett’s multiple comparisons test (*ns* is not significant; *∗p* < 0.05; *∗∗∗p* < 0.001). MD, molecular dynamics.

Class B1 GPCRs are known to bind their endogenous ligands through a multistep binding mechanism (53, 54) where the ECD of the receptor first binds the C terminus of the peptide ligand after which the N terminus of the ligand docks into the TM bundle. First, we investigated whether the tethering of peptide ligands is accommodated by a dynamic ECD that can adopt multiple distinct conformations when the receptor is in the inactive state. By measuring the RMSD of the ECD and root-mean-square fluctuation (RMSF) of each residue in the ECD, we indeed observed that both in the intracellular NAM–bound G protein–free state, and in the ligand- and G protein–free state, the ECD adopted distinct conformations (Fig. 2, *A* and *B*). In contrast to the inactive states, the partial agonist–bound G protein–free state, full agonist–bound G protein–free state, and full agonist and G protein–bound state of the receptor showed reduced flexibility. A comparison of snapshots (Fig. 2*C*) highlighted that the overall 3D architecture of the ECD was stable in all systems. However, in the intracellular NAM–bound G protein–free state, and in the ligand- and G protein–free state of the glucagon receptor, the ECD demonstrated high flexibility in these regions as well as a closing and opening of the orthosteric binding site. This agrees with the previous dynamics studies on ligand-free bound GLP-1R (55), ligand-free and truncated ligands binding in the orthosteric binding site in parathyroid hormone 1 receptor (PTH1R) (56, 57), and ligand-free glucagon receptor in cell membrane and simple bilayers such as POPC and *n*-dodecyl-beta-D-maltopyranoside (47, 58, 59, 60) where in all cases dynamic ECDs were observed.

The increased flexibility in the ECD was seen in three major regions (Fig. 2*B*) on the extracellular side of the ECD with the intracellular NAM–bound G protein–free state and ligand- and G protein–free states being the most dynamic. These three regions were around residue positions T54^ECD^, A73^ECD^, and D103^ECD^. Mutations in and around these residue positions tend to reduce or abolish glucagon-mediated activation of glucagon receptor *in vitro* (61), hence emphasizing their importance experimentally. Altogether, the data highlighted that the ECD is highly dynamic and complement the multistep ligand binding mechanism of class B1 where a ligand’s C terminus seems to be first tethered by the ECD of the glucagon receptor.

### Full agonist and partial agonist show different ligand–receptor interactions in the binding site

**Figure 3:**
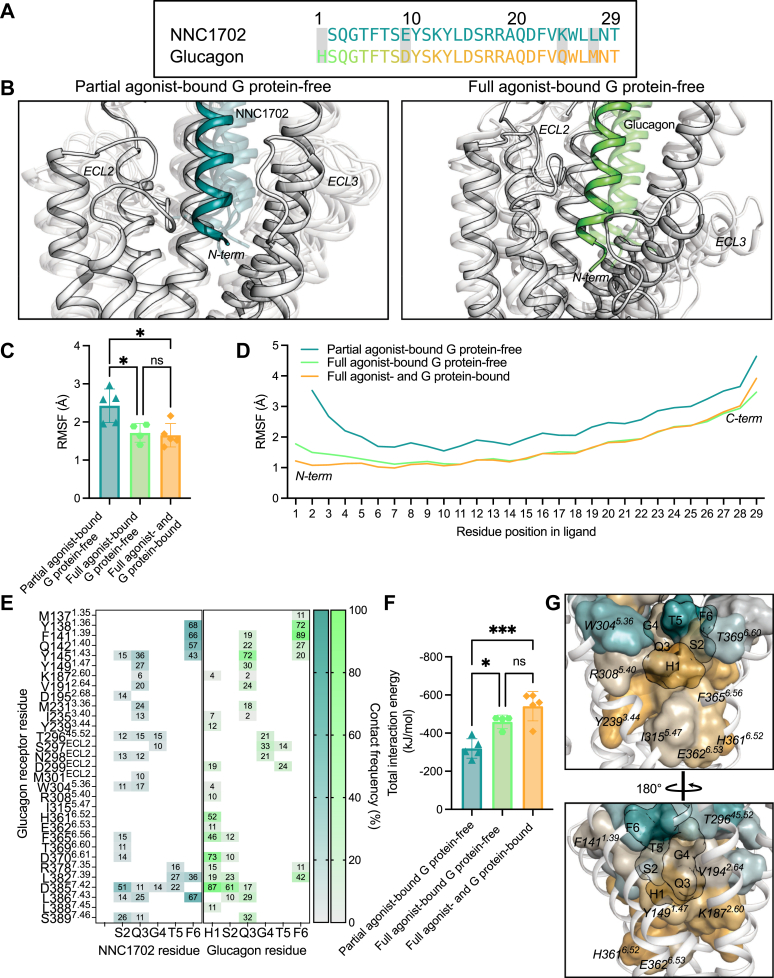
**Ligand–receptor interaction differences between a partial and full agonist on the glucagon receptor.***A*, sequence comparison between NNC1702 and glucagon. *B*, representative snapshots from MD simulations of the ligand-binding site. The snapshots were taken from the unsupervised clustering (RMSD cutoff: 1.2 Ȧ) of the MD ensemble of snapshots (see Table S1 for more details) and then overlayed with the intermediate X-ray structure (Protein Data Bank [PDB] accession number: 5YQZ) and active state cryo-EM structure (PDB accession number: 6LMK), respectively. Root-mean-square fluctuation of (*C*) whole ligand or (*D*) individual residues in each ligand during MD. *E*, N-terminal contact map of NNC1702 and glucagon with the glucagon receptor. *F*, total nonbond interaction energies of N-terminal residues of the NNC1702 (residues 2–6) or glucagon (residues 1–6) with the binding site residues listed in *E*, the interaction energies are comprised of Coulomb and Lennard Jones energies in kJ/mol. *G*, closeup of N-terminal contact differences between NNC1702 and glucagon from the partial agonist–bound G protein–free state and full agonist– and G protein–bound state, respectively. Data represent the mean ± SD of five (four for the full agonist–bound [glucagon] G protein–free state) independent simulation replicates in *C* and *F*, whereas they represent the mean in *D*, statistical significance was assessed using a one-way ordinary ANOVA with Dunnett's multiple comparisons test (*ns* is not significant; *∗p* < 0.05; *∗∗∗p* < 0.001).

As the affinity of the partial agonist, NNC1702, is comparable to that of glucagon in the glucagon receptor experimentally (47), we compared the ligand receptor residue interaction frequency from our MD simulations. As seen in Figure 3*A*, the amino acids in position 1 (deletion *versus* Ser), 9 (Glu *versus* Asp), 24 (Lys *versus* Gln), and 27 (Leu *versus* Met) are different between glucagon and NNC1702. It is also known that removal of the first five amino acids in glucagon leads to antagonism, thus underlying the importance of the N terminus of the peptides in class B1 GPCRs (62).

The partial agonist was more flexible in its binding site in the partial agonist–bound G protein–free state compared with the full agonist in the full agonist–bound G protein–free state as shown in Figure 3*B*. The presence of the G protein further decreased the flexibility of the full agonist (Fig. S3*A*). The MD ensemble of conformations of partial agonist–bound G protein–free state showed five dominant conformation clusters compared with three in the full agonist–bound G protein–free state and one dominant cluster for the full agonist– and G protein–bound (Table S1). Comparing the flexibility using RMSF between the two peptide hormones, confirmed that the partial agonist is more dynamic than the full agonist, both in the G protein–free and G protein–bound states (Fig. 3*C*). Analysis of individual residues of the peptide hormones showed that discrepancies arise from differences in RMSF of the N terminus of both the partial agonist and full agonist (Fig. 3*D*). Molecular analysis of interactions between receptor and ligand indicated that the His in position 1 in glucagon made polar contacts with D370^6.61^ and D385^7.42^ that was absent in NNC1702 (Fig. 3*E*). Y239^3.44^ also made a polar contact in the full agonist–bound G protein–free state; however, the contact frequency with glucagon was even higher when the G protein was present (Fig. S3*B*). In addition, the N terminus of the partial agonist also demonstrated weaker interactions with TM1, TM2, TM3, ECL2, TM5, TM6, and TM7 compared with the full agonist, which was more apparent with the data supported from MD simulations as opposed to the static 3D experimental structures (Figs. 3*E*, S3, *B* and *C* and S4, and Table S2). Mutations in Y239^3.44^ and D385^7.42^ displayed reduced binding of glucagon to the glucagon receptor (63, 64). Ultimately, this allowed for a wider and more open binding pocket of the partial agonist compared with the full agonist, which was confirmed by the interaction energies of the N terminus of the ligands with the receptor (Fig. 3, *F* and *G*). Again, the coupling of the G protein resulted in slightly higher interaction energies of the N terminus of glucagon with its receptor in the active state, suggesting that the G protein allosterically stabilizes the ligand binding in the extracellular region even further.

Overall, these differences in binding site interaction pattern may explain why NNC1702 acts as a partial agonist, whereas glucagon acts as a full agonist on the glucagon receptor, underlining the importance of the N terminus of an orthosteric ligand for receptor allostery and activation. Furthermore, as proteins are not static but undergo conformational rearrangements, MD simulations appear to provide the missing link in explaining differentials in terms of spatial temporal interactions, which often cannot be achieved with data from static 3D experimental structures.

### Repacking of conserved class B1 microswitches upon activation of the glucagon receptor

**Figure 4:**
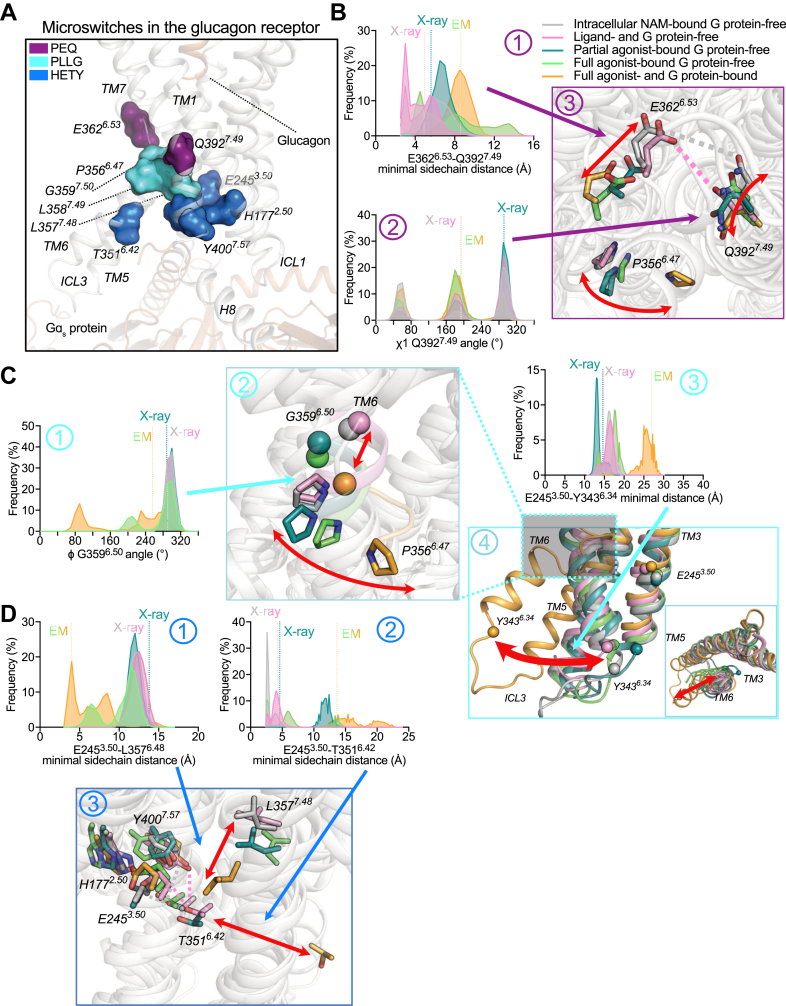
**Repacking of microswitches in the glucagon receptor**. *A*, 3D representation of the P^6.47^Y(E)^6.53^Q^7.49^, P^6.47^x(L)^6.48^x(L)^6.49^G^6.50^, and H^2.50^E^3.50^T^6.42^Y^7.57^ microswitches in the glucagon-bound glucagon receptor in complex with Gɑ_s_ EM structure (Protein Data Bank accession number: 6LMK). *B*, population density plot of minimal distance in any side-chain atoms between the residues E362^6.53^ and Q392^7.49^ (1), population density plot of χ1 angle in Q392^7.49^ (2), and closeup view of P^6.47^Y(E)^6.53^Q^7.49^ microswitch in the glucagon receptor, highlighting dynamics among the different states of the receptor (3). *C*, population density plot of ɸ angle in P356^6.47^ (1), closeup view of P^6.47^x(L)^6.48^x(L)^6.49^G^6.50^ microswitch in the glucagon receptor, highlighting dynamics among the different states of the receptor (2), population density plot of minimal distance in Cɑ atoms between the residues E245^3.50^ and Y343^6.34^ measuring TM3–TM6 distance transition (3), and closeup view of TM6 outward movement between inactive, intermediate, and active states of the receptor (4). *D*, population density plot of minimal distance in any side-chain atoms between the residues E245^3.50^ and L357^6.48^ (1), population density plot of minimal distance in any side-chain atoms between the residues E245^3.50^ and T351^6.42^ (2), and closeup view of H^2.50^E^3.50^T^6.42^Y^7.57^ microswitch in the glucagon receptor, highlighting dynamics among the different states of the receptor (3). Representative snapshots were taken from the top cluster after performing unsupervised clustering (RMSD cutoff: 0.85 Ȧ) of the MD ensemble of snapshots (see Table S3 for more details). Data represent five (four for the full agonist–bound [glucagon] G protein–free state) independent simulation replicates.

To gain insight into the mechanism of activation of the glucagon receptor, we compared the previously identified conserved microswitches in class B1 GPCRs (48, 49): P^6.47^Y(E)^6.53^Q^7.49^, P^6.47^x(L)^6.48^x(L)^6.49^G^6.50^, and H^2.50^E^3.50^T^6.42^Y^7.57^ (Fig. 4 and Table S3).

The conserved P^6.47^Y(E)^6.53^Q^7.49^ motif is located in the middle of TM6 and TM7 in the glucagon receptor (Fig. 4*A*). During MD simulations, we observed that this microswitch is less tight in terms of distances measured between these residues in the partial agonist–bound G protein–free state and full agonist–bound G protein–free state compared to the inactive states. In the full agonist– and G protein–bound state (i.e., active state), the microswitch is disengaged (less tightly packed) even more. This occurrence is characterized by the fact that E362^6.53^ moved away from Q392^7.49^ (Fig. 4*B*-1) as well as increased flexibility of Q392^7.49^ (Fig. 4, *B*-2 and *B*-3). This is in stark contrast with what is observed for the PTH1R (48). In PTH1R, this motif is described as an active microswitch (*i.e.*, distance decreases between residues upon activation) for this receptor. As we thought these discrepancies may be a result of the chosen 3D experimental structures for this study, we pointed toward all the available (a total of 11) 3D structures of the glucagon receptor (45, 46, 47, 51, 52, 63, 65, 66, 67). Here, we noted a similar observation to our MD simulations except for the peptide-20–bound glucagon receptor (PDB accession number: 7V35) and ZP3780-bound glucagon receptor (PDB accession number: 6WPW), which was similar to the intermediate state of the receptor (Fig. S5*A*). The residues that are part of this microswitch showed different side-chain orientations among these structures (Fig. S5*B*). The full agonist– and G protein–bound state MD simulations showed increased contact frequency of E362^6.53^ with H361^6.52^, whereas the intracellular NAM–bound G protein–free state and in the ligand- and G protein–free state, partial agonist–bound G protein–free state, and full agonist–bound G protein–free state showed higher contact frequency of E362^6.53^ with Y239^3.44^ and L358^6.49^ (Fig. S5*C*). Smaller changes were also observed between the different states; however, these were considered minor since the frequency for these contacts was below 40%. Interestingly, mutations in E362^6.53^ are known to abolish binding of glucagon (63). All in all, this suggests that this motif might act differently compared with other class B1 GPCRs.

The conserved motif in class B1 GPCRs that shows typical rearrangement upon activation is the P^6.47^x(L)^6.48^x(L)^6.49^G^6.50^ motif and is located on TM6 just below the P^6.47^Y(E)^6.53^Q^7.49^ motif and right above the H^2.50^E^3.50^T^6.42^Y^7.57^ motif (Fig. 4*A*). Transitioning from the inactive states toward the active states, the dihedral angle (in particular the ɸ angle) of G359^6.50^ rotation caused a kink in TM6 making P356^6.47^ swing out and relocating L357^6.48^ and L358^6.49^ to form a new network of interactions with L242^3.47^, N318^5.50^, F322^5.54^, I355^6.46^, and V396^7.53^ (Figs. 4*C*-1, *C*-2 and S5*C*). Consequently, the TM3–TM6 distance as measured by the distance between E245^3.50^ and Y343^6.34^ increased (Fig. 4*C*-3 and *C*-4). Overall, the P^6.47^x(L)^6.48^x(L)^6.49^G^6.50^ motif demonstrates repacking upon activation in line with other class B1 GPCRs.

The residues that are part of the H^2.50^E^3.50^T^6.42^Y^7.57^ microswitch, which is located on TM2, TM3, TM6, and TM7 and resides below the P^6.47^x(L)^6.48^x(L)^6.49^G^6.50^ motif, showed a increase in distances between them upon activation (Fig. 4*A*). Due to the rearrangement of the P^6.47^x(L)^6.48^x(L)^6.49^G^6.50^ motif and predominantly the relocation of L357^6.48^ toward E245^3.50^ (Fig. 4*D*-1) as well as the loss of interaction of T351^6.42^ with E245^3.50^, L249^3.54^, L354^6.45^, I355^6.46^, and Y400^7.57^ (Fig. 4*D*-2), the H^2.50^E^3.50^T^6.42^Y^7.57^ motif collapsed allowing for the intracellular cavity to open up for the G protein (Figs. 4*D*-3 and S5*C*). Noteworthy, were signatures are even visible in the partial agonist–bound (NNC1702) G protein–free intermediate state of the glucagon receptor during MD, and but were not captured in the static 3D structure. Hence, the activation mechanism of the glucagon receptor is complemented by a set of conserved allosteric microswitches, which either break or form when transitioning from the inactive to active states of the receptor. In line with the P^6.47^x(L)^6.48^x(L)^6.49^G^6.50^ motif, the H^2.50^E^3.50^T^6.42^Y^7.57^ microswitch presents repacking similar to other class B1 GPCRs (48, 49).

### The spatiotemporal persistence of the residue interactions in the glucagon receptor G protein interface reveals hotspots

**Figure 5:**
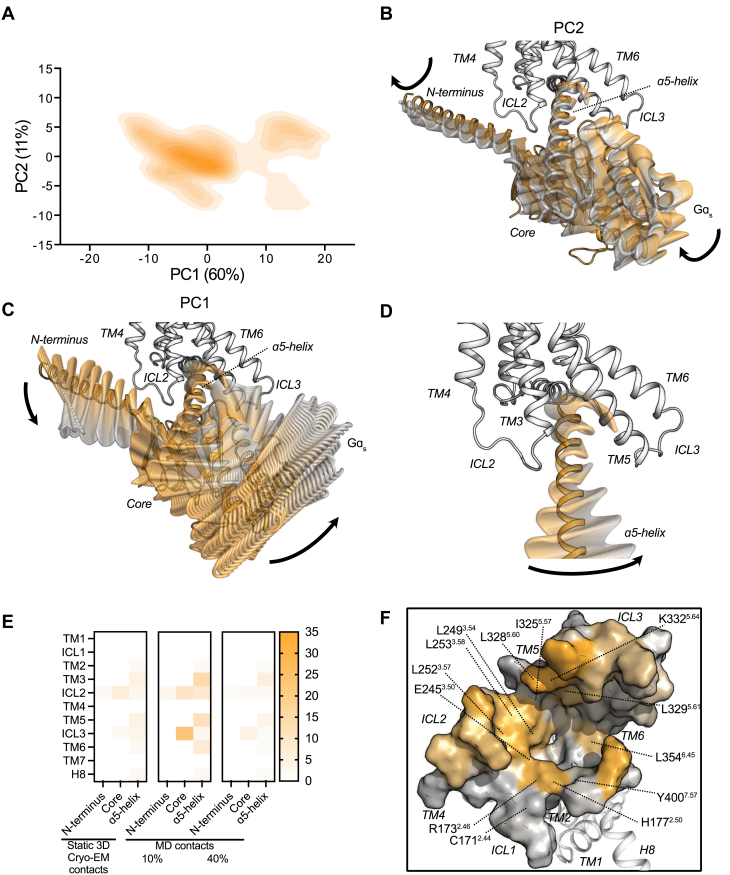
**The G protein interface of the glucagon receptor is dynamic.***A*, principal component (PC) analysis of Gɑ_s_ dynamics consisting of the first (60%) and second (11%) PCs performed on Gɑ_s_'s Cɑ atoms. (*B*) PC1 and (*C*) PC2 representation of Gɑ_s_ portrayed onto the glucagon receptor experimental structure. The PC movement is illustrated using 30 representative snapshots taken from GROMACS function *gmx anaeig*. *D*, ɑ5-helix movement shown from PCs. *E*, segment-based receptor–Gɑ_s_ pairs identified through contact analysis. *F*, G protein interface of the glucagon receptor overlayed with experimentally validated positions that affect G protein signaling. Color gradients indicate low (*gray*) to high (*orange*) contact frequency of receptor residue with G protein. Data represent five independent simulation replicates.

In this section, we seek to uncover the dynamics of the Gα_s_ protein and map the hotspot residue interactions in the glucagon receptor–G protein interface (GPI), which involves both the C terminus α5 helix and the core of the Gα_s_ protein. First, we performed principal component analysis on the MD simulation trajectories of the full agonist and G protein–bound state, to understand the major domain motion in Gα_s_ (Fig. 5, *A*–*C*). The principal component 1 depicted an overall translational movement of Gα_s_ with respect to the receptor, in which the N terminus and core of the G protein rotated away from its starting position (Fig. 5*C*), whereas principal component 2 highlighted a rotation of Gα_s_ around its own axis (Fig. 5*B*). In both cases, the ɑ5-helix displayed a concomitant rearrangement (Fig. 5*D*). Fig. S6 shows all representative conformations of Gα_s_ from the clustering ensemble (Table S4). Altogether, the engagement of Gα_s_ with the glucagon receptor is a spatial and temporal process covering the N terminus, core, and ɑ5-helix interface.

Expanding on these findings, we analyzed the overall interactions between the receptor and Gα_s_ (receptor–Gα_s_ pairs) from both the static 3D experimental structure and the MD simulations. The static 3D structure showed 35 residue contacts involving 21 distinct residues from the glucagon receptor and 19 residues from the Gα_s_. Many of these GPI contacts were weakened during the MD simulations that resulted only in 24 persistent contacts present in greater than 40% of the MD snapshots. There were 89 contacts involving 30 distinct residues from the glucagon receptor and 36 residues from the Gα_s_ with 10% or higher frequency (Figs. 5, *E* and *F*, S7 and Table S5). This exemplified the highly dynamic nature of the G protein (Fig. 5, *B*–*D*). This dynamic behavior is most dominantly observed in the intracellular loops 2 and 3 (ICL2 and ICL3) because of their low contact frequency of interaction with Gα_s_. Gα_s_ N-terminal contacts were mostly formed with ICL1 and ICL2, whereas ICL3 was dominantly interacting with the core of Gα_s_.

Besides the spatiotemporal analysis of the GPI, the interaction energy calculations between the glucagon receptor and G protein residue contacts showed that many of the spatiotemporal interactions in the interface have weak interaction energy (Fig. S8), especially in the core and N terminus of the G protein. On the other hand, the highest interaction energies were found in the α5-helix with the following residues showing the strongest high interaction energy: K332^5.64^ with D381^G.H5.13^ (G protein numbering in superscript (68)) and K405^8.48^ with E392^G.H5.24^. Mutations of the residues C171^2.44^, R173^2.46^, H177^2.50^, E245^3.50^, L249^3.54^, L252^3.57^, L253^3.58^, I325^5.57^, L328^5.60^, L329^5.61^, K332^5.64^, L354^6.45^, and Y400^7.57^ (Fig. 5*F*) showed reduced activation of the glucagon receptor through Gα_s_ (52, 61). The hotspots in the ICLs as well as other newly identified positions in the GPI may also reduce G protein signaling upon mutation. For instance, substituting a positively charged amino acid with a hydrophobic or negatively charged amino acid, or *vice versa*, could alter the coupling efficiency of the G protein to the glucagon receptor. However, the impact of such substitutions on receptor function remains uncertain, and further experiments are warranted to test this hypothesis. Overall, the MD simulations reveal the dynamic nature of the G protein with respect to the receptor.

### SNP residue positions participate in allosteric communication in the glucagon receptor

**Figure 6:**
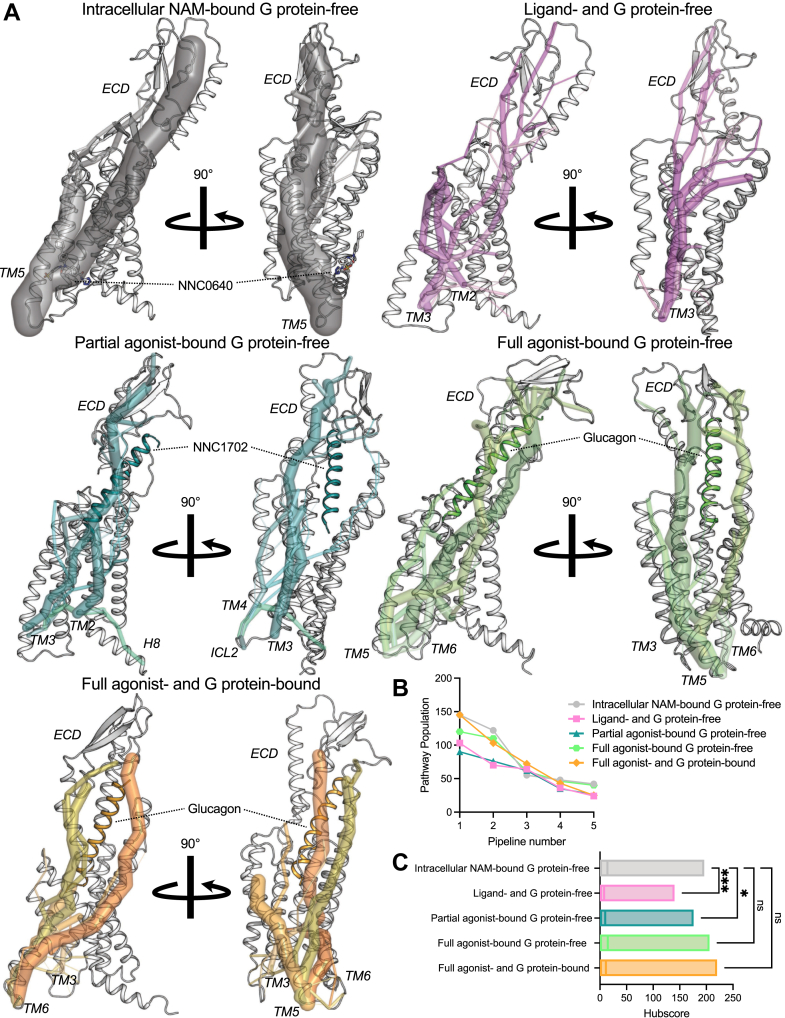
**Allosteric communication in the glucagon receptor.***A*, allosteric communication from the Gα_s_ coupling interface residues to various structural regions in the glucagon receptor displayed on the MD starting structure for each state. The *lines* represent the allosteric communication pipelines with the darkest color having the highest pathway population. *B*, pathway population for top five allosteric pipelines (see Fig. S6 for more details). *C*, distribution of hub scores along the allosteric communication pathways for the different glucagon receptor states. Statistical significance was assessed using a two-way ANOVA with Dunnett's multiple comparisons test (*ns* is not significant; *∗p* < 0.05; *∗∗∗p* < 0.001; between the intracellular NAM–bound G protein–free state and others). Data represent five (four for the full agonist–bound [glucagon] G protein–free state) independent simulation replicates.

Although the allosteric communication mechanisms are well studied in class A GPCRs, such information is sparse for class B1 GPCRs. Therefore, we sought to investigate the allosteric communication in the glucagon receptor from the Gα_s_ coupling interface residues to various structural regions in the receptor (Fig. 6). We used “*Allosteer*” to calculate allosteric communication pipelines in the glucagon receptor between the residues in the Gα_s_ coupling interface and distant residues in the receptor. Briefly, *Allosteer* (69, 70, 71) uses the MD simulation trajectories to calculate the correlation in the torsion angle distribution between pairs of distant residues. Subsequently, it calculates the allosteric communication pathways between distant residues that show high correlated motion. A “hub score” is calculated for each residue in the protein, which is the number of allosteric communication pathways passing through the residue. The allosteric communication pathways are clustered into allosteric communication pipelines. For more details, see the Experimental procedures section. The prediction of residues that play an important role in allosteric communication has been tested out experimentally *via* point mutations in several GPCRs (70) and other proteins (72, 73, 74). Using *Allosteer* on the MD simulation trajectories of various states of the glucagon receptor showed that the allosteric communication from the G protein coupling site stretched beyond the orthosteric ligand–binding site (Fig. 6). More specifically, the allosteric communication in any of the receptor states reached the ECD of the glucagon receptor (Fig. 6), although with different communication strengths, suggesting that the ECD allosterically communicates with the GPI in different functional states.

The top five allosteric communication pipelines showed communication from the ECD to TM5 in intracellular NAM–bound G protein–free state (Figs. 6, *A* and *B* and S9). Removing the NAM weakened the allosteric communication dramatically and moved the top five allosteric communication pipelines from the ECD to TM2 and TM3. The presence of the partial agonist increased the overall allosteric communication, and the top five pipelines stretched from the ECD all the way to TM2, TM3, ICL2, TM4, and H8. In the full agonist–bound G protein–free state and full agonist– and G protein–bound state, the allosteric communication was enhanced more than in the partial agonist–bound G protein–free state, having the top five communication pipelines transitioning between the ECD and TM3, TM5, and TM6. These findings were confirmed by examining the allosteric hub score, which is a measure of the number of pathways that go through a residue (Fig. 6*C*). Taken together, these results show that the allosteric communication is stronger in the intracellular NAM–bound G protein–free state, full agonist– and G protein–bound state and the full agonist–bound and G protein–free state compared with the partial agonist–bound G protein–free and ligand– and G protein–free state, with the latter being the weakest in allosteric communication.

**Figure 7:**
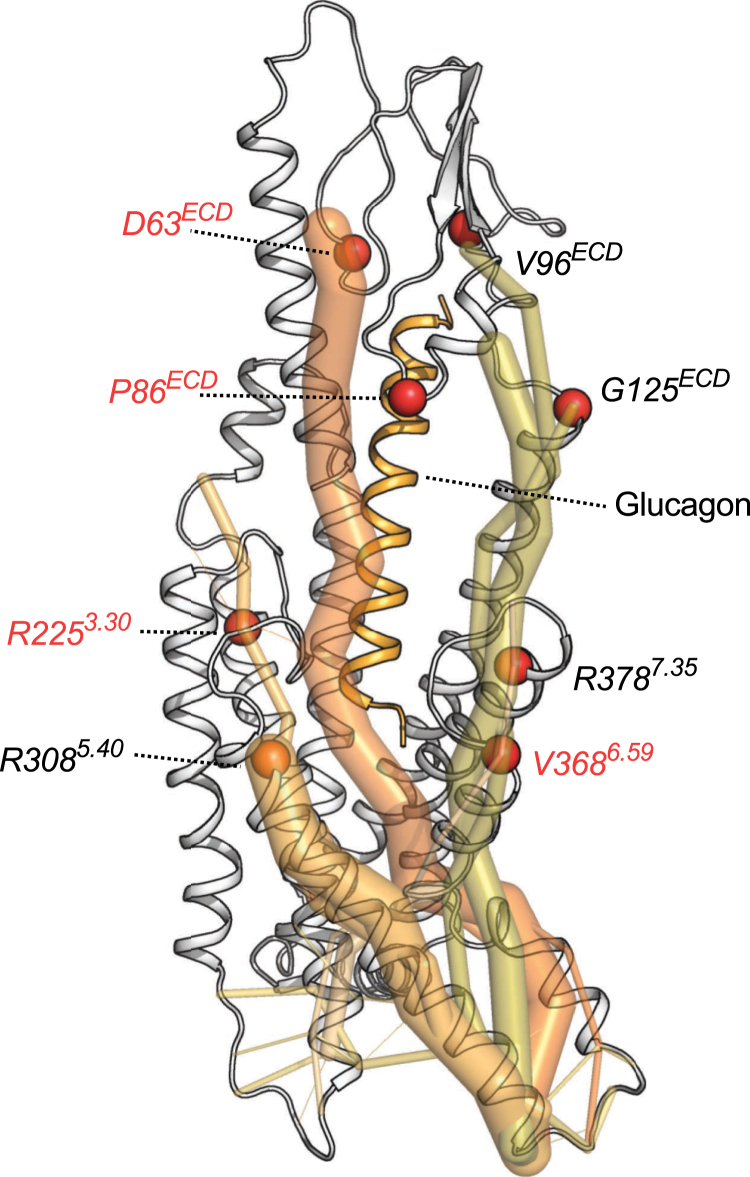
**SNP positions are part of the allosteric communication in the glucagon receptor.** SNP locations and the allostery are portrayed on the full agonist–(glucagon) and G protein–bound receptor state, with the G protein removed from the final figure rendering. *Red spheres* indicate that these SNP positions have been associated with disease traits. SNP data were taken from the study by van der Velden *et al.* (25). Data represent five independent simulation replicates.

Previously, it was identified that SNPs in the glucagon receptor can result in decreased cAMP accumulation through decreased Gɑ_s_ coupling *in vitro* (25). As some of these may directly be associated to be part of the glucagon binding site (<5 Ȧ), some were more distantly located (>5 Ȧ). One may say that these positions of which SNPs exist are located directly in the allosteric communication pipelines. To investigate this hypothesis, we explored the allosteric communication and in particular the hub scores more closely for the following SNP positions that showed decreased G protein coupling *in vitro*: D63^ECD^, P86^ECD^, V96^ECD^, G125^ECD^, R225^3.30^, R308^5.40^, V368^6.59^, and R378^7.35^ (Fig. 7). Upon inspection of the major allosteric communication pipelines, it became evident that the positions D63^ECD^, V96^ECD^, G125^ECD^, R225^3.30^, R308^5.40^, V368^6.59^, and R378^7.35^ were indeed part of the allosteric communication in the glucagon receptor. On top of that, P86^ECD^ did seem to be partially important for allosteric communication but was also located next to W87^ECD^, which was directly interacting with glucagon and hence may be part of the second shell of interactions with the ligand (Fig. S3*C*). Interestingly, three of the four SNP positions with clinically disease-associated traits for the SNPs D63^ECD^N, R225^3.30^H, and V368^6.59^M had among the higher hub scores in the glucagon receptor, suggesting that the strength of the hub score may reflect the importance of being potentially disease associated.

## Discussion

The glucagon receptor is an important drug target for diabetes and metabolic disorders because of its involvement in metabolism. Finding novel drugs targeting this receptor and in combination with GLP-1R is pivotal to combat these metabolic diseases. The present study shed light on how the dynamics and allostery are regulated within the glucagon receptor using a variety of *in silico* techniques. For instance, we observed that the ECD is highly dynamic, and that the allosteric communication mechanism from the GPI can reach all the way to the ECD in the receptor.

Although several SNPs are documented in the glucagon receptor, the functional annotation of these SNPs is not available. This is because these SNPs are not necessarily located in the orthosteric ligand–binding site or at the G protein coupling sites. The role of SNPs that are allosteric to both these functional sites in the glucagon receptor is unknown. Here, we showed that the residue positions with high frequency of SNPs often are involved in mediating the allosteric communication between the ECD and the G protein coupling interface. Furthermore, we highlighted that disease-associated SNPs are important hubs in allosteric communication to the G protein. These findings lead to therapeutic strategies to identify putative binding sites near the SNPs to target the disease-associated mutants specifically. Hence, these findings may aid in the design of future small-molecule allosteric modulators for the glucagon receptor.

Collectively, peptides are the cognate orthosteric ligands for class B1 GPCRs. Drug discovery attempts for class B1 often tend to focus on the orthosteric binding sites first by targeting the receptors with peptide mimetics (75, 76). Allosteric antagonists have been presented for the glucagon receptor (77, 78), including two that were crystalized with the glucagon receptor and are in the intracellular region (46, 47). As current efforts from the community have focused on the design of antagonists, the therapeutic effects of positive small-molecule allosteric modulators have been overlooked. The therapeutic effects of glucagon receptor agonists seem to be more promising, especially in combination with GLP-1R agonism, since chronic administration of water-soluble glucagon leads to improved glucose tolerance, decreased bodyweight, lower food intake, increased lipid metabolism, and enhanced energy expenditure in mice (79).

Since experimental structures only provide us with one snapshot of the overall complex, MD simulations can contribute significantly to the dynamics of a receptor and thus explore unidentified features of such system. To our knowledge, this is the first MD study to include the whole ensemble of different functional states (*i.e.*, inactive, intermediate, and active states) of the glucagon receptor; however, some groups have explored the activation mechanism by exploiting several of these functional states (60, 80, 81). Consequently, we were able to capture the domain motions in the ECD, TMs, loop regions, ligand dynamics, extensive repacking of microswitches, the rotational and translational movements of the G protein toward the receptor, as well as how the allosteric communication is transmitted through the glucagon receptor. Previously, MD simulations have provided insight in the mechanism of coupling of Gɑ_s_ to the glucagon receptor (82); however, the spatiotemporal information and dynamic range of the G protein with respect to the glucagon receptor were unknown.

As we know from the static structure of the glucagon receptor with G protein that the ICLs are responsible for the G protein coupling and particularly for Gɑ_s_ recognition (52). In addition, it has been established that ICL3 is directly involved into the blockage and opening of the G protein binding cavity in class A GPCRs through conformational equilibrium (83), something which is also seen for the C terminus (84). The residue contact analysis in the GPI substantiated the assertion of the importance of ICLs in the glucagon receptor. More interestingly, our work further elaborated on these statements by accentuating the importance of the core residues on top of the ɑ5-helix residues in the G protein that contact the glucagon receptor’s ICLs, in particular ICL2 and ICL3. These data may show why certain G protein–receptor pairs might act differently upon mutation of the receptor’s GPI. Altogether, this information provided us with additional sets of G protein–receptor pairs, in which future experimental research is warranted to uncover their exact impact on G protein coupling.

We have calculated the allosteric communication in the glucagon receptor using the *Allosteer* method. *Allosteer* is an information theoretic approach that uses MD simulation trajectories to calculate the ensemble of allosteric communication pipelines between distant structural regions in proteins (69, 71, 85, 86, 87). *Allosteer* has been used for the prediction of residues involved in allosteric communication and validated with subsequent experimental testing through mutations of residues that are distant from the active sites by measuring protein function through FRET, bioluminescence resonance energy transfer, and cell-based secondary messenger measurements for several GPCRs (70, 88, 89, 90, 91, 92), G proteins (93), kinases (72, 94), phosphatases (74), DNA repair proteins (73), as well as for calculating bias factors of ligands for GPCRs (89, 92). In this study, we have validated the residues predicted by our method using mutations that affect glucagon receptor function that were previously unexplained.

In case of the glucagon receptor, the allosteric communication is weak in the ligand– and G protein–free state. The allosteric communication is stronger in the partial agonist–bound G protein–free state, full agonist–bound G protein–free state, full agonist– and G protein–bound state, and intracellular NAM–bound G protein–free state. These findings agree with data observed in the β2-adrenergic receptor (69) where the allosteric communication in the inactive state was also stronger compared with the active state. Interestingly, the top allosteric pipelines presented us with unique allosteric communication pathways emanating from the GPI to various structural regions in the receptor including the ECD. Mutations in the residue positions that are part of these allosteric pipelines, such as TM5 and TM6, may affect G protein coupling and could bias signaling toward β-arrestins, especially when changed to amino acid residues with different side-chain properties, which was observed previously in our work on the angiotensin II receptor 1 (70).

In conclusion, we demonstrated the highly dynamic behavior of the glucagon receptor, which shows key conformational signatures when transitioning from the inactive to the active state, and thereby providing novel insights on how the glucagon receptor’s allostery functions. The provided framework in the present study as such can be harnessed to start the design of allosteric modulators for the glucagon receptor. This may include the exploration of allosteric binding sites that are connected to the present allosteric pipelines and or located near SNP positions in the glucagon receptor.

## Experimental procedures

### Structure selection, model building, and system building

The following 3D experimental glucagon receptor structures were utilized in this study: glucagon receptor–NNC0640–mAb1 complex (PDB accession number: 5XEZ) (47), glucagon receptor–NNC1702–T4 complex (PDB accession number: 5YQZ) (51) and glucagon receptor–glucagon–Gɑ_s_–Gβ_1_–Gγ_2_–NB35 complex (PDB accession number: 6LMK) (52). Ligand– and G protein–free state model was created based on the 5XEZ structure by removing the NAM and the antibody. The intracellular NAM–bound (NNC0640) G protein–free state model was generated based on the 5XEZ structure by only removing the antibody. The partial agonist–bound (NNC1702) G protein–free state model was created by removing the T4 lysozyme from the 5YQZ complex. The full agonist (glucagon) and G protein–bound (Gɑ_s_–Gβ_1_–Gγ_2_) state model of the glucagon receptor bound was generated based on the 6LMK structure by extracting the NB35. The full agonist–bound (glucagon) G protein–free state model was generated using the 6LMK structure by removing the NB35 and Gɑ_s_–Gβ_1_–Gγ_2_. All mutations in the 3D experimental structures were reverted to wildtype, and missing domains/loops were generated using the Prime module within Schrödinger Maestro (Prime, Schrödinger, LLC, 2022; Maestro, Schrödinger, LLC, 2022) (95, 96), except for the alpha helical domain in Gɑ_s_. Next, the missing side chains as well as hydrogen atoms were added, and histidine-protonated states were assigned. All models were energy minimized using the standard settings in the protein preparation wizard module (Prime, Schrödinger, LLC, 2022) (97).

### MD simulation setup

The MD simulation boxes for each of the systems were generated using Chemistry at HARvard Molecular Mechanics (CHARMM) graphical user interface (98). The orientation of proteins in membranes database (https://opm.phar.umich.edu) was used to position the glucagon receptor in the membrane bilayer (99), after which a pre-equilibrated POPC bilayer was generated. The final system dimensions were as follows: intracellular NAM–bound (NNC0640) G protein–free state model 100 × 100 × 146, including 258 lipids, 31,874 waters, and 150 mM NaCl; ligand– and G protein–free state model 100 × 100 × 146 A, including 258 lipids, 31,907 waters, and 150 mM NaCl; partial agonist–bound (NNC1702) G protein–free state model 100 × 100 × 149 A, including 258 lipids, 32,570 waters, and 150 mM NaCl; full agonist–bound (glucagon) G protein–free state model 100 × 100 × 151 A, including 258 lipids, 33,433 waters, and 150 mM NaCl; full agonist (glucagon) and G protein–bound (Gɑ_s_–Gβ_1_–Gγ_2_) state model 120 × 120 × 207 A, including 386 lipids, 71,465 waters, and 150 mM NaCl. We ran the MD simulations using the GROningen MAchine for Chemical Simulations (GROMACS) package (version 2022) (100, 101) with the CHARMM36 forcefield for proteins, POPC lipids, ions, and using CHARMM Transferable Intermolecular Potential with 3 Points water as solvent. First, we minimized each system after which it was equilibrated using a constant temperature, constant volume (NVT) ensemble (1 ns), and consequently with a constant temperature, constant pressure (NPT) ensemble (gradually reduced position restraints from 5 to 0 kcal/mol/A^2^ and each 5 ns long). The final step of the equilibration involved a 100 ns unrestrained NPT ensemble. Next, the final snapshot of the equilibration step served as the initial conformation for a total of five production MD simulations with different starting velocities carried out each 1 μs long, with the simulation snapshots stored every 20 ps and the integration step being 2 fs. For the full agonist–bound (glucagon) G protein–free state model, we ran 5 μs long unbiased MD simulations to allow for transition toward the intermediate state. The system pressure was set to 1 bar pressure bath and controlled using the Parrinello–Rahman method. The LINCS algorithm was applied to all bonds and angles of water molecules. Finally, a 12 Å cutoff was used for nonbonded interactions, and the particle mesh Ewald method treated the long-range L–J interactions.

### Data analysis

The entire 1 μs∗5 runs translating to 5 μs simulation time was utilized for all analyses, except for the full agonist–bound (glucagon) G protein–free state model where the last 1.5 μs of four runs translating to 6 μs simulation time was used since the RMSD of the TM backbone and glucagon backbone were most stable during this simulation time. Run 2 was omitted since it did not transition toward the intermediate state within the 5 μs simulation time. Trajectories were visualized using Visual Molecular Dynamics (102) and PyMOL (The PyMOL Molecular Graphics System, version 2.0; Schrödinger, LLC), and basic analysis was done using the GROMACS analysis package (100, 101). Data are presented as mean ± SD unless stated otherwise in the legend. To assess the convergence of the MD simulations, the RMSD of TM backbone was calculated using *gmx rms* using the following glucagon receptor residues: TM1: 132 to 165, TM2: 173 to 199, TM3: 221 to 254, TM4: 264 to 288, TM5: 305 to 334, TM6: 343 to 367, and TM7: 377 to 400 (Fig. S2, *A*–*E*). The RMSD of the NNC0640, NNC1702 backbone, glucagon backbone (Fig. S2, *A*, *C*, *D*, and *E*), Gɑ_s_, Gβ, and Gγ were also calculated using the gmx rms function (Fig. S2, *F*–*H*). To obtain the representative snapshots from MD simulations, we used *gmx cluster* to calculate the most populated cluster representatives for receptor or ligand conformations at different RMSD cutoffs (Tables S1, S3, and S4). The principal component analysis on the Gɑ_s_ was done on the Cɑ atoms using *gmx covar* to obtain all eigenvectors and subsequently *gmx anaeig* to portray the first two eigenvectors. The corresponding snapshots of principal component 1 and principal component 2 were obtained using the -extr and -nframes of 30 function in *gmx anaeig*.

### Calculation of ligand–receptor interactions and receptor–G protein interactions

To calculate contact frequency between the contacts made by different amino acids in the GPCR:GPI and also in the ligand–receptor interface during our MD simulations, we used the python script named *Get_Contacts* (https://getcontacts.github.io/). The contact frequency is defined as the percentage of MD snapshots that contain a specific contact. The contact frequency cutoff between the ligand and receptor and receptor G protein was set to 40% (corresponding to at least two simulation runs) or stated otherwise. No cutoff was used for microswitch contact frequency. Frequencies of ligand–receptor, receptor–G protein interactions, and microswitches were displayed as a heatmap using GraphPad Prism, version 10 (GraphPad Software, Inc).

### Calculation of allosteric communication pipelines using Allosteer

Our inhouse allosteric communication method (“*Allosteer*”) was utilized to calculate the allostery in the glucagon receptor. In short, it uses the MD trajectories to calculate the allosteric communication pipelines and to identify the network of residues involved in allosteric communication (69, 70). First, the method calculates the correlation in torsion angle distribution for each pair of residues, which is called the mutual information (MI). For residue pairs that were >10 Å and with MI values in the top 10% of all MI, we used the shortest path algorithm by Dijkstra (103) which is implemented in MATLAB (The MathWorks, Inc [2020; MATLAB version: 9.13.0 [R2020]; https://www.mathworks.com) to compute the shortest pathway with maximum MI. The nodes in the pathways represented residues with high MI but does not necessarily require any type of direct interaction between them since they may or may not be neighbors. Following this, the pathways were clustered into pipelines based on their mutual proximity in the receptor structure. Each residue in the glucagon receptor has several allosteric pathways passing through the residue, which is the hub score for that residue. The residues that contribute to the allosteric communication pipelines modulate the couplings strength of the G protein by the glucagon receptor.

### Statistical analysis

For our computational analysis, statistical significance was addressed using a one-way ordinary ANOVA with Dunnett's multiple comparisons test. The definition of statistical significance was *p* < 0.05.

## Data availability

Our codes are available upon request here: https://www.cityofhope.org/research/beckman-research-institute/computational-and-quantitative-medicine/vaidehi-lab/vaidehi-lab-software. Or upon reasonable request: NVaidehi@coh.org. All data are present in either the main text or the supporting information.

## Supporting information

This article contains supporting information.

## Conflict of interest

The authors declare that they have no conflicts of interest with the contents of this article.
