# Supplementary material for: Allosteric communication mechanism in the glucagon receptor
Source: J Biol Chem. 2025 Apr 23;301(6):108530. doi: 10.1016/j.jbc.2025.108530 (PMC12145835; doi:10.1016/j.jbc.2025.108530)
Supplement: Supplementary data [file mmc1.docx]

Supplementary information:

Allosteric Communication Mechanism in the Glucagon Receptor

Wijnand J. C. van der Velden^1,2,*^, Elizaveta Mukhaleva^1^ and Nagarajan Vaidehi^1*^

^1^ Department of Computational and Quantitative Medicine, Beckman Research Institute of the City of Hope, Duarte, CA 91010, USA.

^2^ Present address: Aikium Inc., 2630 Bancroft Way, Berkeley, CA 94704, USA

^*^ Corresponding author: [nvaidehi@coh.org](mailto:nvaidehi@coh.org); wijnandvandervelden@me.com

# Running title

Allosteric communication in the glucagon receptor

# Keywords

Glucagon, Glucagon receptor, G protein-coupled receptor (GPCR), molecular dynamics, allosteric communication, single nucleotide polymorphism, class B1, G protein, diabetes, metabolic disorders

# Supplementary Tables

## Table S1 | Unsupervised clustering of the MD ensemble of snapshots. The aggregated trajectories of each system were clustered by applying the single linkage method on the ligand backbone atoms. A root-mean-squared deviation cutoff of 1.2 Å was used for ligand backbone clustering. The representative structures of the clusters that together add up to >89% of the population are shown below. The remainder population (i.e., to get a 100% of the MD snapshots) for each system was distributed among the remaining clusters.

|  | Percentage of simulation frames in each conformational cluster | | |
| --- | --- | --- | --- |
| Cluster (number) | Partial agonist-bound G protein-free | Full agonist-bound G protein-free | Full agonist- and G protein-bound |
| 1 | 57 | 49 | 100 |
| 2 | 15 | 25 |  |
| 3 | 6 | 25 |  |
| 4 | 6 |  |  |
| 5 | 5 |  |  |

## Table S2 | Differences in glucagon receptor positions that interact with the full agonist or partial agonist between the static experimental structure and from MD. Cutoff 10%.

| Partial agonist-bound G protein-free MD | Partial agonist-bound G protein-free Experimental (5YQZ) | Full agonist- and G protein-bound MD | Full agonist- and G protein-bound Experimental (6LMK) | Full agonist-bound G protein-free |
| --- | --- | --- | --- | --- |
| 27 | 27 | 27 | 27 | 27 |
| 28 | 28 | 28 | 28 | 28 |
| 29 | 29 | 29 | 29 | 29 |
|  |  |  |  | 30 |
| 32 | 32 | 32 | 32 | 32 |
| 33 | 33 | 33 | 33 | 33 |
| 36 | 36 | 36 |  | 36 |
| 63 |  |  |  | 63 |
| 64 | 64 | 64 | 64 | 64 |
| 65 | 65 | 65 | 65 | 65 |
|  |  |  |  | 81 |
|  |  |  |  | 82 |
| 84 | 84 | 84 | 84 | 84 |
| 85 | 85 | 85 |  | 85 |
| 87 | 87 | 87 |  | 87 |
|  |  |  |  | 96 |
|  |  |  |  | 98 |
|  |  | 111 |  |  |
| 113 |  | 113 |  | 113 |
|  |  |  |  | 114 |
|  |  |  |  | 115 |
| 116 | 116 | 116 | 116 | 116 |
|  |  |  |  | 117 |
| 118 | 118 | 118 |  | 118 |
|  |  | 121 |  | 121 |
| 122 |  | 122 |  | 122 |
| 123 | 123 | 123 | 123 | 123 |
|  |  |  |  | 124 |
|  |  |  |  | 125 |
|  |  |  |  | 126 |
|  |  |  |  | 127 |
| 128 |  |  |  |  |
| 131 | 131 | 131 | 131 | 131 |
|  |  |  |  | 132 |
| 134 | 134 | 134 | 134 | 134 |
| 135 | 135 | 135 | 135 | 135 |
|  |  |  |  | 137 |
| 138 | 138 | 138 | 138 | 138 |
| 139 |  |  |  | 139 |
| 141 | 141 | 141 | 141 | 141 |
| 142 | 142 | 142 | 142 | 142 |
| 145 | 145 | 145 | 145 | 145 |
| 149 | 149 | 149 | 149 | 149 |
|  |  | 187 |  |  |
| 191 | 191 | 191 | 191 | 191 |
|  | 194 | 194 |  |  |
| 195 | 195 | 195 |  |  |
| 198 | 198 | 198 | 198 | 198 |
| 199 |  | 199 |  | 199 |
|  |  |  | 201 | 201 |
| 202 | 202 | 202 | 202 | 202 |
|  |  | 203 |  | 203 |
| 204 | 204 | 204 | 204 | 204 |
|  | 205 | 205 |  | 205 |
| 206 | 206 | 206 | 206 | 206 |
| 207 | 207 | 207 | 207 | 207 |
| 208 | 208 | 208 |  | 208 |
| 209 | 209 | 209 |  | 209 |
|  |  |  |  | 210 |
|  |  |  |  | 211 |
| 212 |  | 212 | 212 | 212 |
| 215 | 215 | 215 | 215 | 215 |
|  |  |  |  | 216 |
| 231 |  | 231 | 231 | 231 |
| 235 |  | 235 | 235 |  |
|  |  | 239 |  | 239 |
|  | 293 |  | 293 |  |
| 296 | 296 | 296 | 296 | 296 |
| 297 | 297 | 297 | 297 | 297 |
| 298 | 298 | 298 | 298 | 298 |
| 299 | 299 | 299 |  | 299 |
| 301 |  |  |  |  |
| 304 |  |  | 304 |  |
|  |  | 308 |  | 308 |
|  |  |  | 311 |  |
|  |  | 315 |  |  |
|  |  | 361 | 361 | 361 |
|  |  | 362 |  | 362 |
| 365 |  | 365 | 365 | 365 |
| 369 | 369 |  |  |  |
| 370 |  | 370 | 370 | 370 |
|  |  |  |  | 372 |
|  | 374 |  |  |  |
| 378 | 378 | 378 | 378 | 378 |
|  |  |  | 381 |  |
| 382 | 382 | 382 | 382 | 382 |
| 385 | 385 | 385 | 385 | 385 |
| 386 | 386 | 386 | 386 | 386 |
|  |  |  |  | 388 |
| 389 |  | 389 |  | 389 |

## Table S3 | Unsupervised clustering of the MD ensemble of snapshots. The aggregated trajectories of each system were clustered by applying the single linkage method on the TM backbone atoms. A root-mean-squared deviation cutoff of 0.85 Å was used for TM backbone clustering. The representative structures of the clusters that together add up to >89% of the population are shown below. The remainder population (i.e., to get a 100% of the MD snapshots) for each system was distributed among the remaining clusters.

|  |  | | Percentage of simulation frames in each conformational cluster | | | |
| --- | --- | --- | --- | --- | --- | --- |
| Cluster (number) | Intracellular NAM-bound (NNC0640) G protein-free | Ligand- and G protein-free state | | Partial agonist-bound G protein-free | Full agonist-bound G protein-free | Full agonist- and G protein-bound |
| 1 | 84 | 77 | | 87 | 25 | 50 |
| 2 | 7 | 10 | | 12 | 25 | 18 |
| 3 |  | 4 | |  | 25 | 9 |
| 4 |  | 4 | |  | 25 | 6 |
| 5 |  |  | |  |  | 5 |

## Table S4 | Unsupervised clustering of the MD ensemble of snapshots. The aggregated trajectories of each system were clustered by applying the single linkage method on the G protein backbone atoms. A root-mean-squared deviation cutoff of 2.6 Å was used for G protein backbone clustering. The representative structures of the clusters that together add up to >89% of the population are shown below. The remainder population (i.e., to get a 100% of the MD snapshots) for each system was distributed among the remaining clusters.

|  | Percentage of simulation frames in each conformational cluster |
| --- | --- |
| Cluster (number) | Full agonist- and G protein-bound |
| 1 | 60 |
| 2 | 17 |
| 3 | 12 |

## Table S5 | Differences in glucagon receptor contacts that interact with Gɑ_s_ between EM and MD. The contact frequency cutoff for MD was 10%.

| Full agonist- and G protein-bound MD  Contact frequency | Full agonist- and G protein-bound Experimental (6LMK)  Contact Yes or No |
| --- | --- |
| 173 | 173 |
|  | 177 |
| 245 | 245 |
| 248 | 248 |
| 249 | 249 |
| 252 | 252 |
| 253 | 253 |
| 254 |  |
| 255 |  |
| 256 | 256 |
| 257 | 257 |
| 258 | 258 |
| 259 | 259 |
| 260 | 260 |
|  | 261 |
| 325 |  |
| 328 |  |
| 329 | 329 |
| 332 | 332 |
| 333 |  |
| 335 |  |
| 336 | 336 |
| 337 | 337 |
| 338 |  |
| 339 |  |
| 340 |  |
| 343 |  |
| 346 |  |
|  | 351 |
| 354 | 354 |
| 355 |  |
| 404 | 404 |
| 405 | 405 |

# Supplementary Figures

**Figure S1 | Full agonist-bound G protein-free TM3-TM6 transition.** The TM3-TM6 distance was measured as the minimal distance of Cɑ atoms between the residues E245^3.50^ and Y343^6.34^ over the course of 5,000 ns. Inactive and active state TM3-TM6 distance were obtained from the following experimental structures: the intracellular NAM-bound (NNC0640) G protein-free X-ray structure (PDB ID: 5XEZ) and the full agonist (glucagon) and G protein-bound (Gɑ_s_-Gβ_1_-Gγ_2_) EM structure (PDB ID: 6LMK). Data represent five independent simulation replicates.

**Figure S2 | Quality control of MD simulations. (A)** Root-mean-square deviation of the TM-backbone of the glucagon receptor during ligand- and G protein-free inactive state simulations. **(B)** Root-mean-square deviation of TM-backbone of the glucagon receptor during intracellular NAM-bound (NNC0640) G protein-free inactive state, partial agonist-bound (NNC1702) G protein-free intermediate state, full agonist-bound (glucagon) G protein-free intermediate state, and full agonist (glucagon) and G protein-bound (Gɑ_s_-Gβ_1_-Gγ_2_) active state. **(C)** Root-mean-square deviation of ligand or ligand backbone (in the case of orthosteric peptides) of the glucagon receptor during intracellular NAM-bound (NNC0640) G protein-free inactive state, partial agonist-bound (NNC1702) G protein-free intermediate state, full agonist-bound (glucagon) G protein-free intermediate state, and full agonist (glucagon) and G protein-bound (Gɑ_s_-Gβ_1_-Gγ_2_) active state simulations. **(D)** Root-mean-square deviation of Gɑ_s_-backbone during full agonist (glucagon) and G protein-bound (Gɑ_s_-Gβ_1_-Gγ_2_) active state simulations. **(E)** Root-mean-square deviation of Gβ_1_-backbone during full agonist (glucagon) and G protein-bound (Gɑ_s_-Gβ_1_-Gγ_2_) active state simulations. **(F)** Root-mean-square deviation of Gγ_2_-backbone during full agonist (glucagon) and G protein-bound (Gɑ_s_-Gβ_1_-Gγ_2_) active state simulations. Data represent five independent simulation replicates.

**Figure S3 | Glucagon binding pocket in the full agonist (glucagon) and G protein-bound (Gɑs-Gβ1-Gγ2) active state simulations. (A)** Representative snapshots from MD simulations of the ligand binding site. The snapshots were taken from the unsupervised clustering (RMSD cutoff: 1.2 Ȧ) of the MD ensemble of snapshots (see Table S1 for more details), and then overlayed with the active state Cryo-EM structure (PDB ID: 6LMK). **(B)** Ligand-receptor interaction differences between partial and full agonist on the glucagon receptor. **(C)** Contact heatmap of full agonist with the glucagon receptor. Ligand-receptor contact cut-offs were 10%. Data represent five (four for the full agonist-bound (glucagon) G protein-free state) independent simulation replicates.

**Figure S4 | Contact fingerprints of orthosteric agonists with the glucagon receptor in the partial agonist-bound (NNC1702) G protein-free intermediate state and full agonist-bound (glucagon) G protein-free intermediate state.** **(A)** Contact heatmap of partial agonist (NNC1710) with the glucagon receptor. **(B)** Contact heatmap of full agonist (glucagon) with the glucagon receptor. Ligand-receptor contact cut-offs were 10%. Data represent five independent simulation replicates.

**Figure S5 | Microswitches in the glucagon receptor. (A)** PEQ motif distance measured by calculating the minimal distance between any sidechain atoms of E362^6.53^ and Q392^7.49^ from all available 3D experimental structures of the glucagon receptor. **(B)** 3D representation of the P^6.47^Y(E)^6.53^Q^7.49^ microswitch in all available experimental glucagon receptor structures. **(C)** Intramolecular contact heatmaps of important microswitch residues, E362^6.53^, L357^6.48^, L358^6.49^, and T351^6.42^ with other residues in the glucagon receptor among the different simulated systems.

Data represent five (four for the full agonist-bound (glucagon) G protein-free state) independent simulation replicates in **C**.

**Figure S6 | G protein conformational ensemble from MD.** Representative snapshots were taken from the top cluster after performing unsupervised clustering (RMSD cutoff: 2.6 Ȧ) of the MD ensemble of snapshots (see Figure S4 for more details). Data represent five independent simulation replicates.

**Figure S7 | Contact fingerprints of G protein with the glucagon receptor.**

Contact heatmap of G protein residues with the glucagon receptor. G protein-receptor contact cut-offs were 10%. Data represent five independent simulation replicates.

**Figure S8 | Interaction energies of G protein and glucagon receptor pairs.** Interaction contact heatmap of G protein residues with the glucagon receptor. Data represent five independent simulation replicates.

**Figure S9 | Allosteric communication in the glucagon receptor.**

Pathway population for allosteric communication to Gɑ_s_ obtained from Allosteer. Data represent five (four for the full agonist-bound (glucagon) G protein-free state) independent simulation replicates.
