# Supplementary material for: Allosteric communication mechanism in the glucagon receptor
Source: J Biol Chem. 2025 Apr 23;301(6):108530. doi: 10.1016/j.jbc.2025.108530 (PMC12145835; doi:10.1016/j.jbc.2025.108530)
Supplement: Supplementary figures [file mmc2.zip › Supplementary_Figures_resubV1_WJC_withNumbering.pdf]

Sup Fig 1

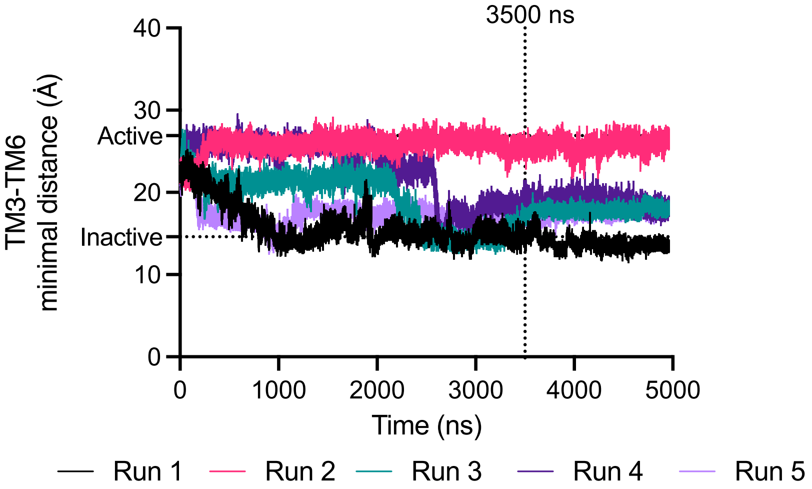
3500 ns
TM3-TM6
minimal distance (Å)
40
30
Active
20
Inactive
10
0
0
1000
2000
3000
4000
5000
Time (ns)
Run 1
Run 2
Run 3
Run 4
Run 5

Sup Fig 2

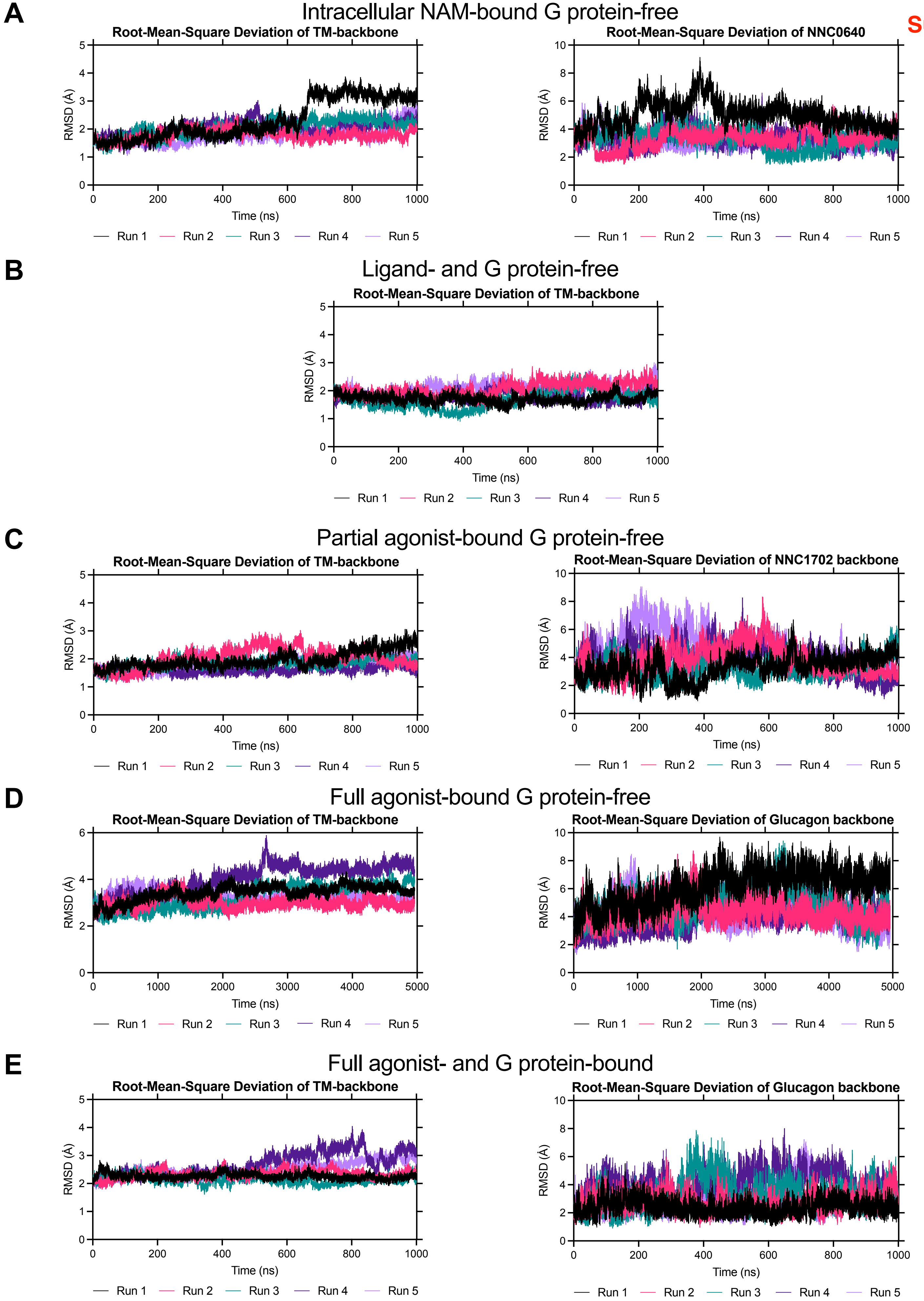
A
Intracellular NAM-bound G protein-free
Root-Mean-Square Deviation of TM-backbone
Root-Mean-Square Deviation of NNC0640
RMSD (Å)
Time (ns)
Run 1
Run 2
Run 3
Run 4
Run 5
B
Ligand- and G protein-free
Root-Mean-Square Deviation of TM-backbone
C
Partial agonist-bound G protein-free
Root-Mean-Square Deviation of TM-backbone
Root-Mean-Square Deviation of NNC1702 backbone
D
Full agonist-bound G protein-free
Root-Mean-Square Deviation of TM-backbone
Root-Mean-Square Deviation of Glucagon backbone
E
Full agonist- and G protein-bound
Root-Mean-Square Deviation of TM-backbone
Root-Mean-Square Deviation of Glucagon backbone

Sup Fig 2

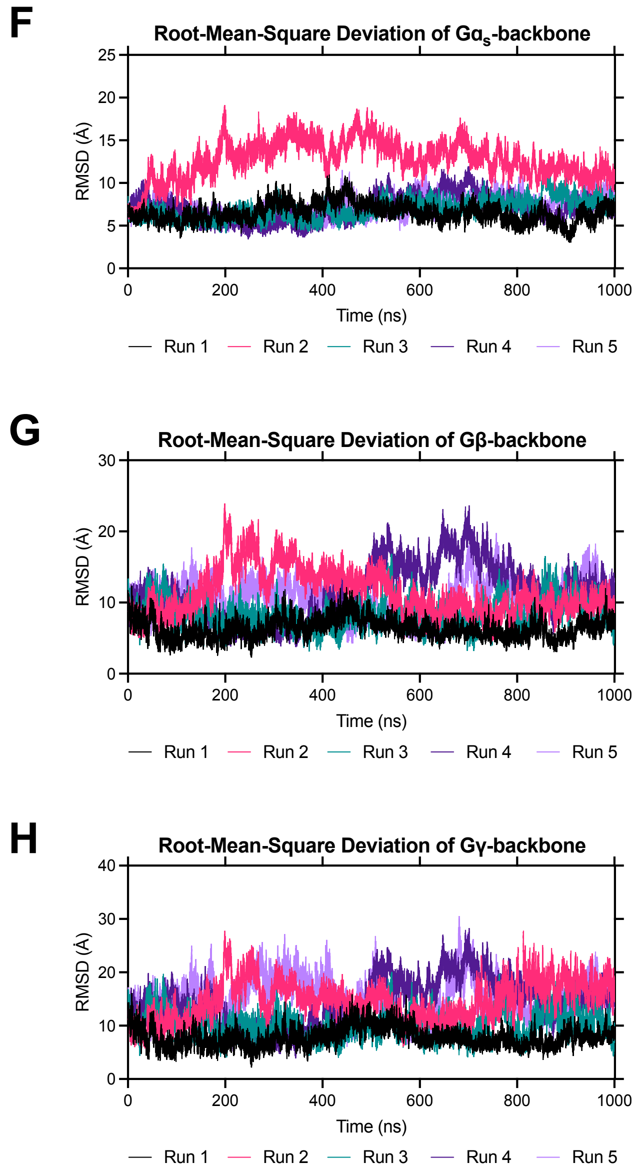
F
Root-Mean-Square Deviation of $G\alpha_s$-backbone
RMSD (Å)
0
5
10
15
20
25
0
200
400
600
800
1000
Time (ns)
Run 1
Run 2
Run 3
Run 4
Run 5
G
Root-Mean-Square Deviation of Gβ-backbone
RMSD (Å)
0
10
20
30
0
200
400
600
800
1000
Time (ns)
Run 1
Run 2
Run 3
Run 4
Run 5
H
Root-Mean-Square Deviation of Gγ-backbone
RMSD (Å)
0
10
20
30
40
0
200
400
600
800
1000
Time (ns)
Run 1
Run 2
Run 3
Run 4
Run 5

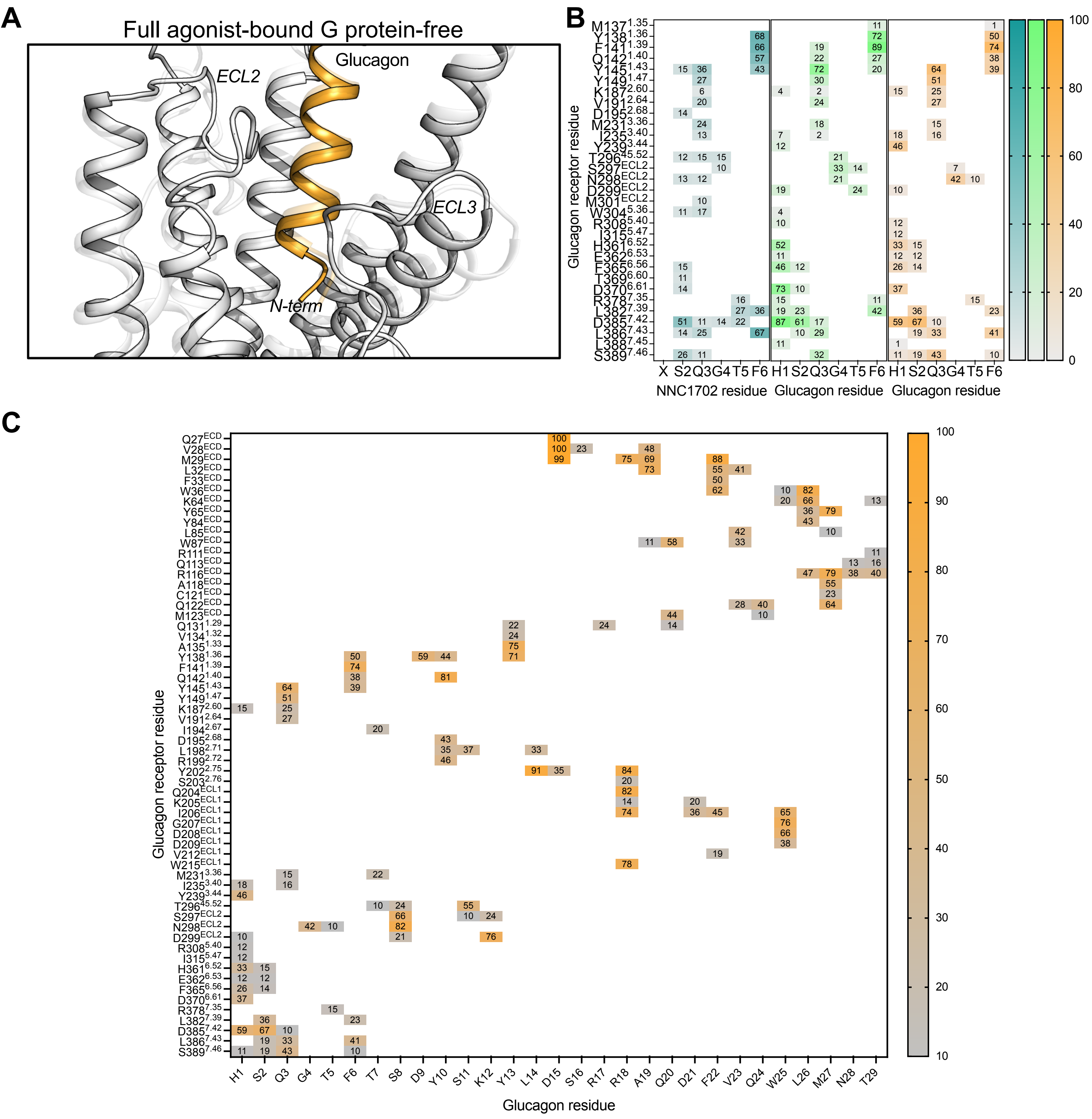
A
Full agonist-bound G protein-free
Glucagon
ECL2
ECL3
N-term
B
Glucagon receptor residue
NNC1702 residue
Glucagon residue
Glucagon residue
C
Glucagon receptor residue
Glucagon residue

Sup Fig 3

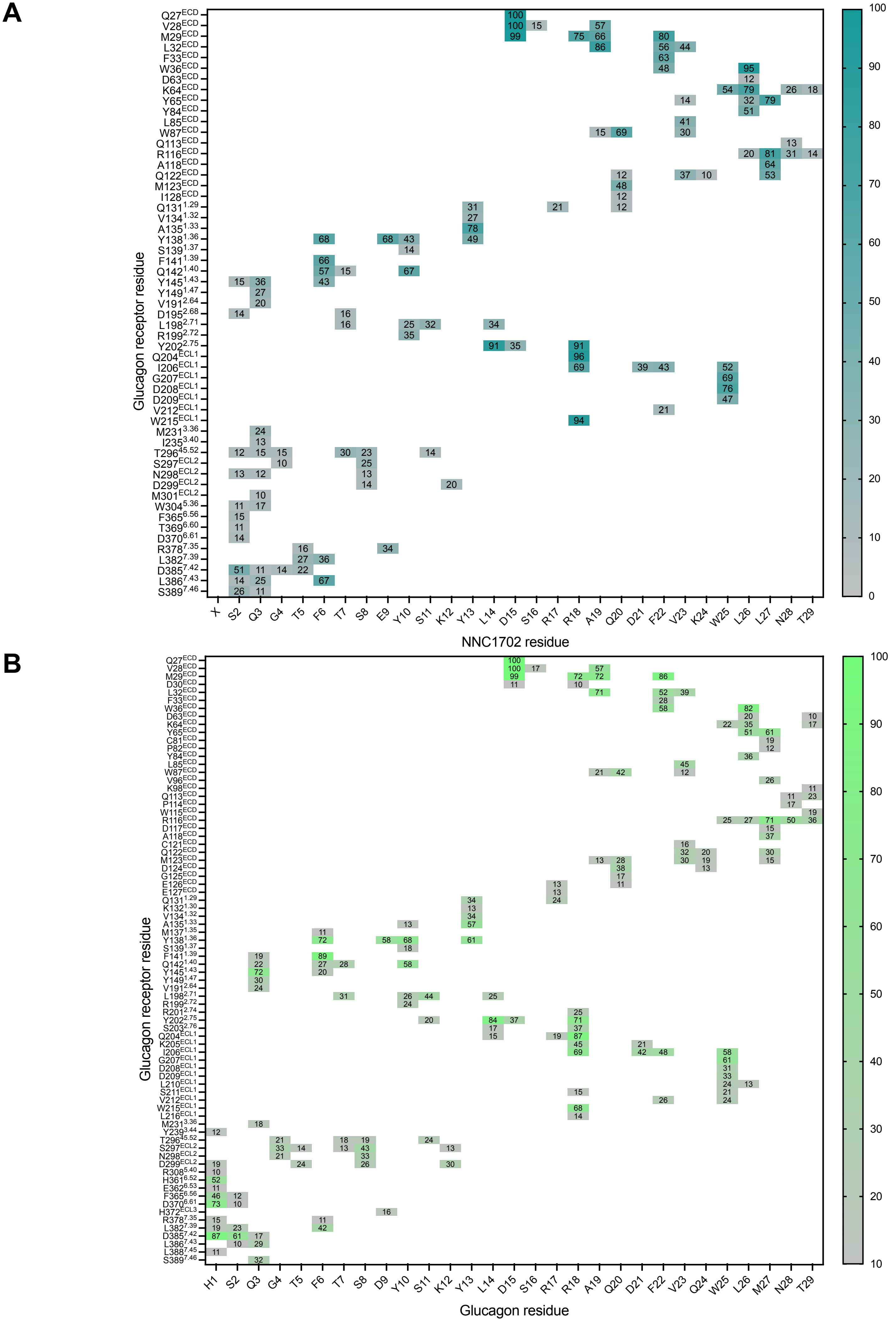
A
Glucagon receptor residue
NNC1702 residue
B
Glucagon receptor residue
Glucagon residue

Sup Fig 4

Sup Fig 5

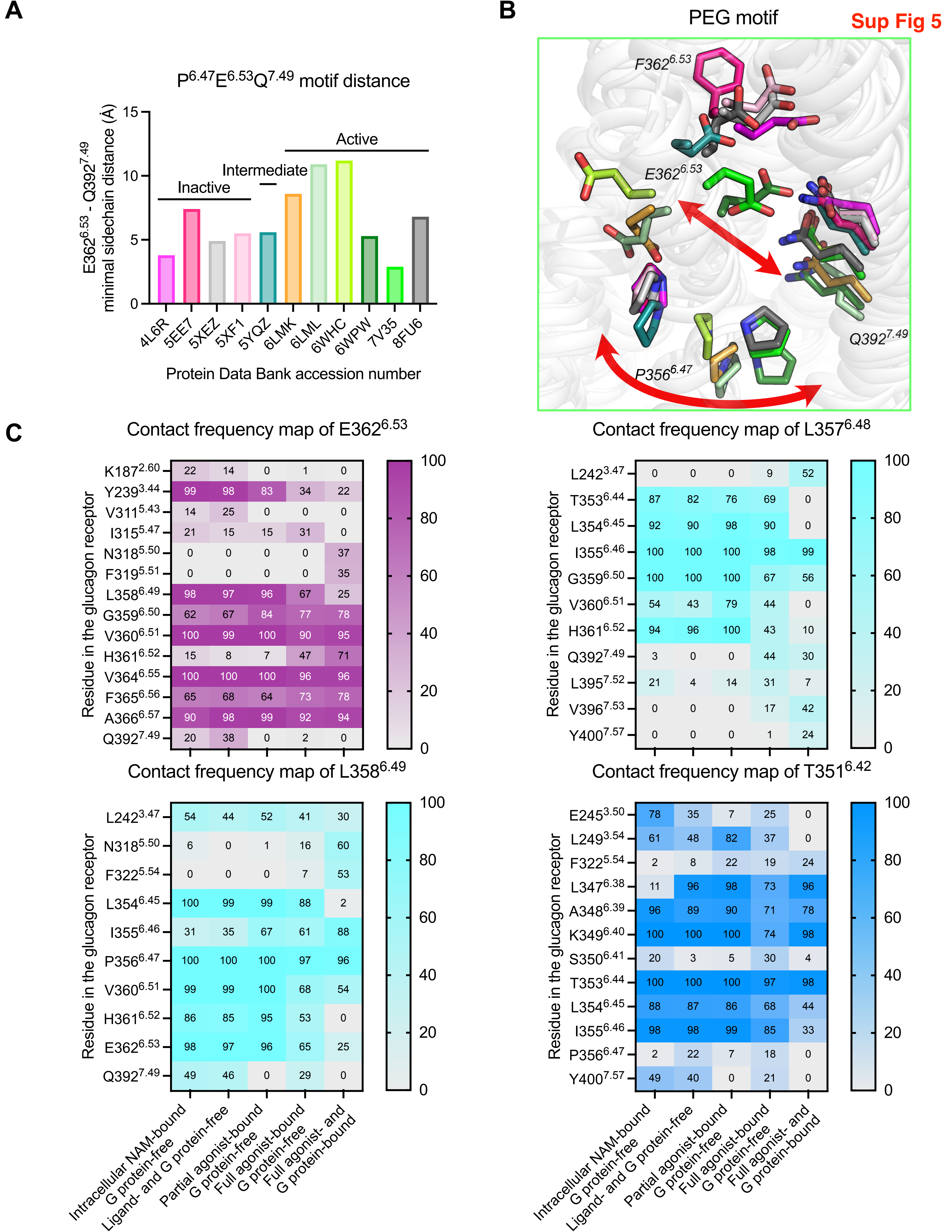
A
P6.47E6.53Q7.49 motif distance
E3626.53 - Q3927.49 minimal sidechain distance (Å)
Inactive
Intermediate
Active
4L6R
5EE7
5XEZ
5XF1
5YQZ
6LMK
6LML
6WHC
6WPW
7V35
8FU6
Protein Data Bank accession number
B
PEG motif
F3626.53
E3626.53
Q3927.49
P3566.47
C
Contact frequency map of E3626.53
Residue in the glucagon receptor
K1872.60 22 14 0 1 0
Y2393.44 99 98 83 34 22
V3115.43 14 25 0 0 0
I3155.47 21 15 15 31 0
N3185.50 0 0 0 0 37
F3195.51 0 0 0 0 35
L3586.49 98 97 96 67 25
G3596.50 62 67 84 77 78
V3606.51 100 99 100 90 95
H3616.52 15 8 7 47 71
V3646.55 100 100 100 96 96
F3656.56 65 68 64 73 78
A3666.57 90 98 99 92 94
Q3927.49 20 38 0 2 0
Contact frequency map of L3576.48
L2423.47 0 0 0 9 52
T3536.44 87 82 76 69 0
L3546.45 92 90 98 90 0
I3556.46 100 100 100 98 99
G3596.50 100 100 100 67 56
V3606.51 54 43 79 44 0
H3616.52 94 96 100 43 10
Q3927.49 3 0 0 44 30
L3957.52 21 4 14 31 7
V3967.53 0 0 0 17 42
Y4007.57 0 0 0 1 24
Contact frequency map of L3586.49
L2423.47 54 44 52 41 30
N3185.50 6 0 1 16 60
F3225.54 0 0 0 7 53
L3546.45 100 99 99 88 2
I3556.46 31 35 67 61 88
P3566.47 100 100 100 97 96
V3606.51 99 99 100 68 54
H3616.52 86 85 95 53 0
E3626.53 98 97 96 65 25
Q3927.49 49 46 0 29 0
Contact frequency map of T3516.42
E2453.50 78 35 7 25 0
L2493.54 61 48 82 37 0
F3225.54 2 8 22 19 24
L3476.38 11 96 98 73 96
A3486.39 96 89 90 71 78
K3496.40 100 100 100 74 98
S3506.41 20 3 5 30 4
T3536.44 100 100 100 97 98
L3546.45 88 87 86 68 44
I3556.46 98 98 99 85 33
P3566.47 2 22 7 18 0
Y4007.57 49 40 0 21 0
Intracellular NAM-bound G protein-free
Ligand- and G protein-free
Partial agonist-bound G protein-free
Full agonist-bound G protein-free
Full agonist- and G protein-bound

Sup Fig 6

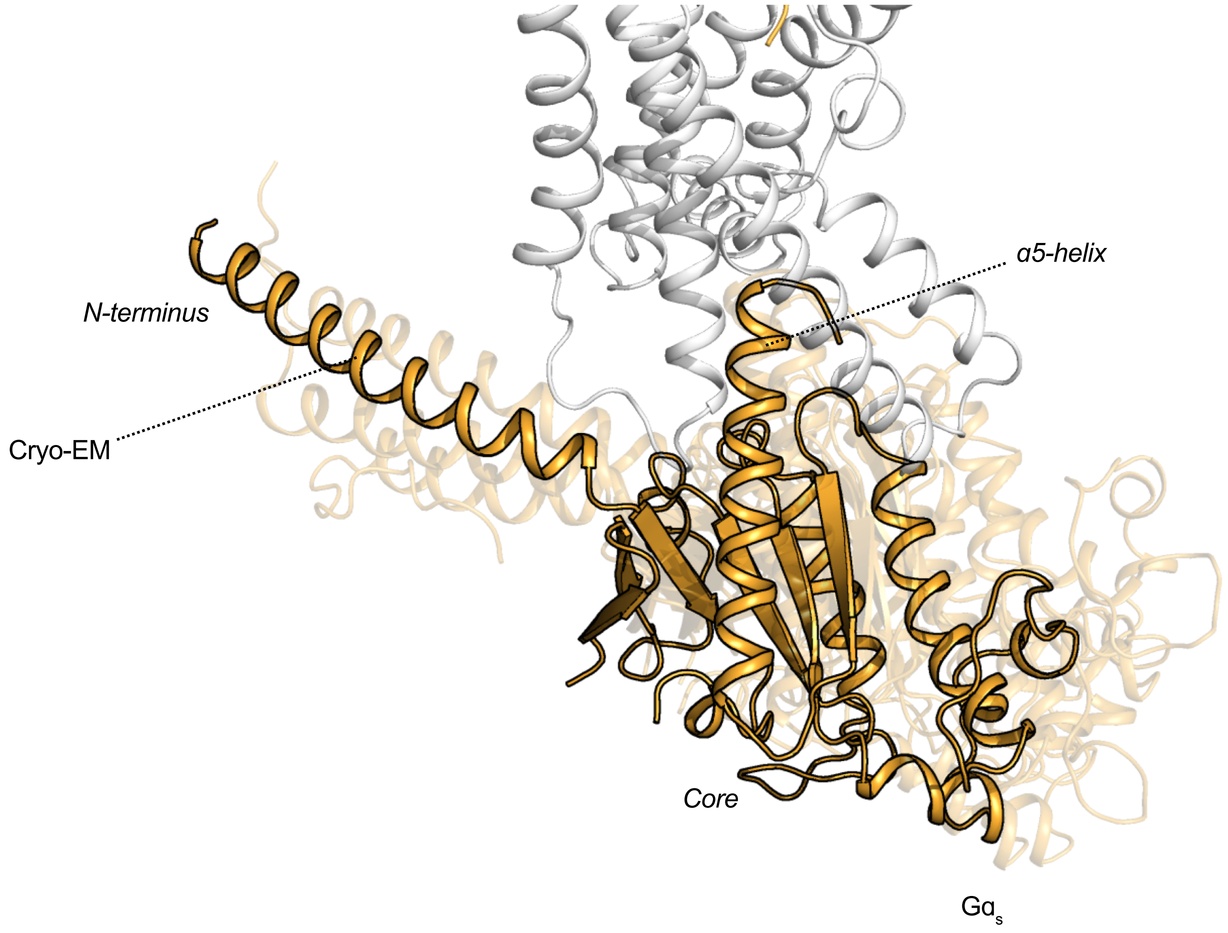

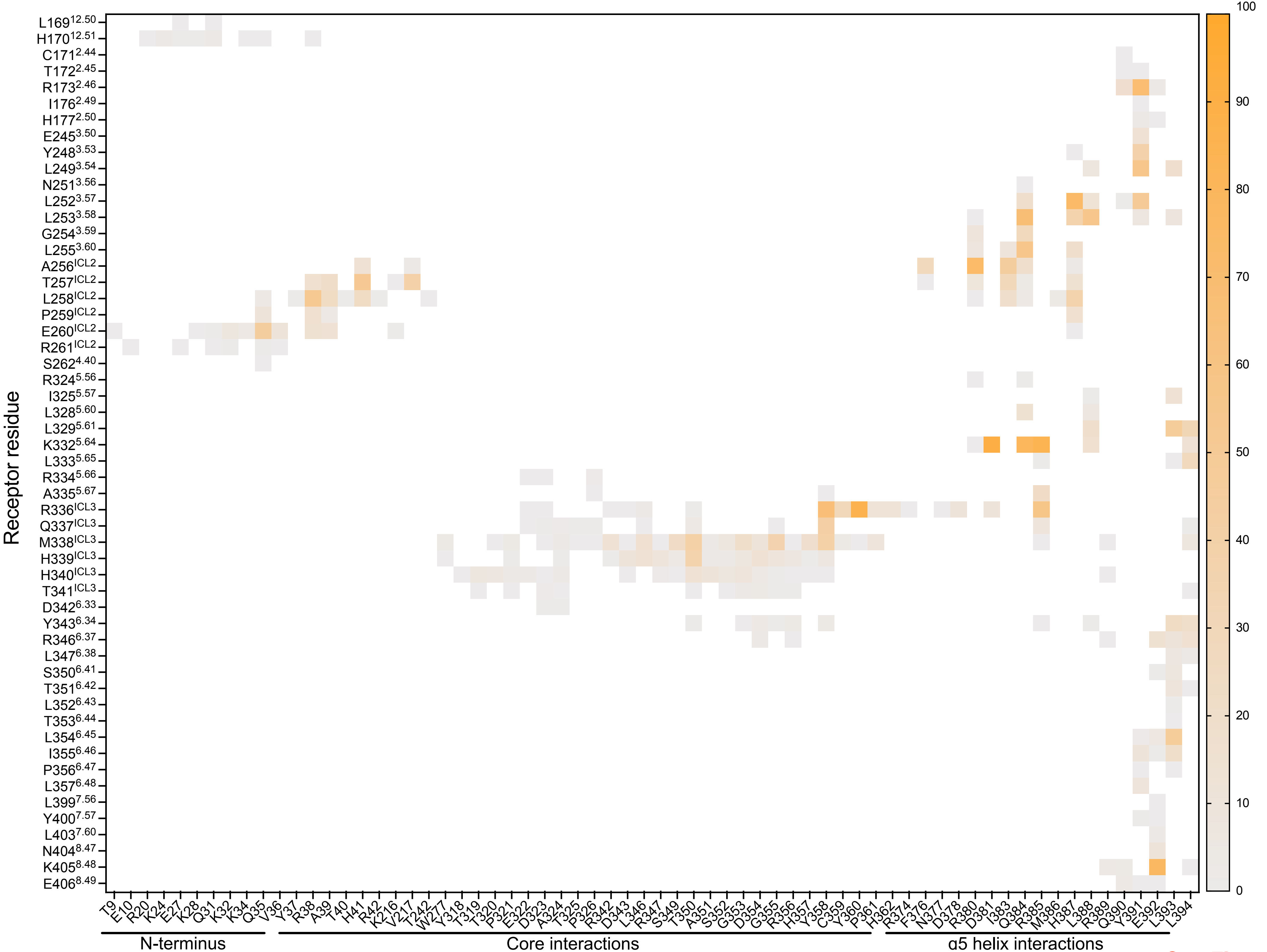
Receptor residue
L169 12.50
H170 12.51
C171 2.44
T172 2.45
R173 2.46
I176 2.49
H177 2.50
E245 3.50
Y248 3.53
L249 3.54
N251 3.56
L252 3.57
L253 3.58
G254 3.59
L255 3.60
A256 ICL2
T257 ICL2
L258 ICL2
P259 ICL2
E260 ICL2
R261 ICL2
S262 4.40
R324 5.56
I325 5.57
L328 5.60
L329 5.61
K332 5.64
L333 5.65
R334 5.66
A335 5.67
R336 ICL3
Q337 ICL3
M338 ICL3
H339 ICL3
H340 ICL3
T341 ICL3
D342 6.33
Y343 6.34
R346 6.37
L347 6.38
S350 6.41
T351 6.42
L352 6.43
T353 6.44
L354 6.45
I355 6.46
P356 6.47
L357 6.48
L399 7.56
Y400 7.57
L403 7.60
N404 8.47
K405 8.48
E406 8.49
T9 E10 R20 K24 E27 K28 Q31 K32 K34 Q35 V36 Y37 R38 A39 T40 H41 R42 K216 V217 T242 W277 Y318 T319 T320 P321 E322 D323 A324 T325 P326 R342 D343 L346 R347 S349 T350 A351 S352 G353 D354 G355 R356 H357 Y358 C359 Y360 P361 H362 R374 F376 N377 D378 R380 D381 I383 Q384 R385 M386 H387 L388 R389 Q390 Y391 E392 L393 L394
N-terminus interactions
Core interactions
α5 helix interactions
Gαs residue
100
90
80
70
60
50
40
30
20
10
0

Sup Fig 7

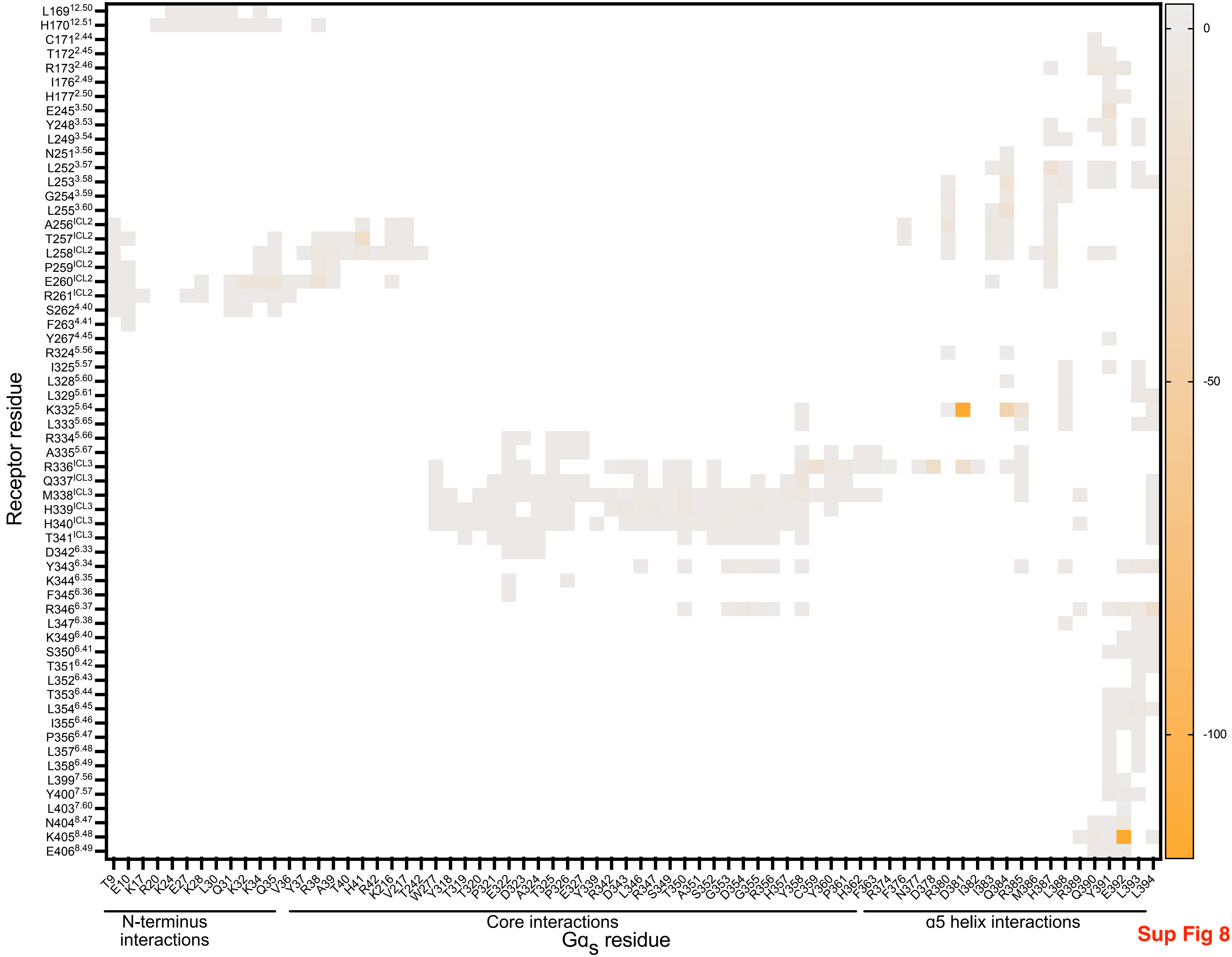
Receptor residue
L169$^{12.50}$
H170$^{12.51}$
C171$^{2.44}$
T172$^{2.45}$
R173$^{2.46}$
I176$^{2.49}$
H177$^{2.50}$
E245$^{3.50}$
Y248$^{3.53}$
L249$^{3.54}$
N251$^{3.56}$
L252$^{3.57}$
L253$^{3.58}$
G254$^{3.59}$
L255$^{3.60}$
A256^{ICL2}
T257^{ICL2}
L258^{ICL2}
P259^{ICL2}
E260^{ICL2}
R261^{ICL2}
S262$^{4.40}$
F263$^{4.41}$
Y267$^{4.45}$
R324$^{5.56}$
I325$^{5.57}$
L328$^{5.60}$
L329$^{5.61}$
K332$^{5.64}$
L333$^{5.65}$
R334$^{5.66}$
A335$^{5.67}$
R336^{ICL3}
Q337^{ICL3}
M338^{ICL3}
H339^{ICL3}
H340^{ICL3}
T341^{ICL3}
D342$^{6.33}$
Y343$^{6.34}$
K344$^{6.35}$
F345$^{6.36}$
R346$^{6.37}$
L347$^{6.38}$
K349$^{6.40}$
S350$^{6.41}$
T351$^{6.42}$
L352$^{6.43}$
T353$^{6.44}$
L354$^{6.45}$
I355$^{6.46}$
P356$^{6.47}$
L357$^{6.48}$
L358$^{6.49}$
L399$^{7.56}$
Y400$^{7.57}$
L403$^{7.60}$
N404$^{8.47}$
K405$^{8.48}$
E406$^{8.49}$
T9 E10 K17 R20 K24 E27 K28 L30 Q31 K32 K34 Q35 V36 Y37 R38 A39 T40 H41 R42 K216 V217 T242 W277 Y318 T319 T320 P321 E322 D323 A324 T325 P326 E327 Y339 R342 D343 L346 R347 S349 T350 A351 S352 G353 D354 G355 R356 H357 Y358 C359 Y360 P361 H362 F363 R374 F376 N377 D378 R380 D381 I382 I383 Q384 R385 M386 H387 L388 R389 Q390 Y391 E392 L393 L394
N-terminus interactions
Core interactions
α5 helix interactions
Gα$_s$ residue
0
-50
-100

Sup Fig 8

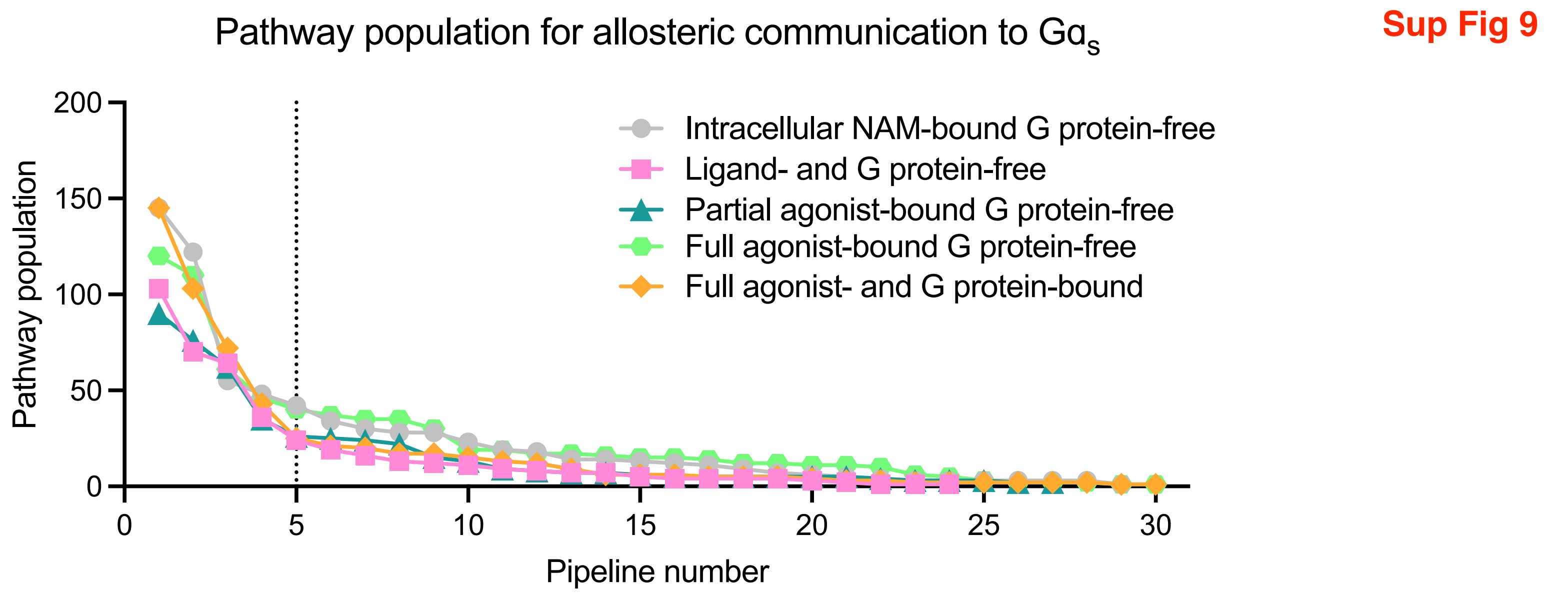
Pathway population for allosteric communication to $G\alpha_s$
Sup Fig 9
Pathway population
Pipeline number
0
50
100
150
200
0
5
10
15
20
25
30
Intracellular NAM-bound G protein-free
Ligand- and G protein-free
Partial agonist-bound G protein-free
Full agonist-bound G protein-free
Full agonist- and G protein-bound
